# Auditory opportunity and visual constraint enabled the evolution of echolocation in bats

**DOI:** 10.1038/s41467-017-02532-x

**Published:** 2018-01-08

**Authors:** Jeneni Thiagavel, Clément Cechetto, Sharlene E. Santana, Lasse Jakobsen, Eric J. Warrant, John M. Ratcliffe

**Affiliations:** 1 Department of Ecology and Evolutionary Biology, University of Toronto, 25 Willcocks Street, Toronto, ON M5S 3B2 Canada; 2 Department of Biology, University of Southern Denmark, Campusvej 55, 5230, Odense C, Denmark; 3 Department of Biology and Burke Museum of Natural History and Culture, University of Washington, Seattle, WA 98195 USA; 4 Department of Biology, Lund University, Sölvegatan 35, 22362 Lund, Sweden; 5 Department of Biology, University of Toronto Mississauga, 3359 Mississauga Road, Mississauga, ON L5L 1C6 Canada; 6 Department of Natural History, Royal Ontario Museum, 100 Queens Park, Toronto, ON M5S 2C6 Canada

## Abstract

Substantial evidence now supports the hypothesis that the common ancestor of bats was nocturnal and capable of both powered flight and laryngeal echolocation. This scenario entails a parallel sensory and biomechanical transition from a nonvolant, vision-reliant mammal to one capable of sonar and flight. Here we consider anatomical constraints and opportunities that led to a sonar rather than vision-based solution. We show that bats’ common ancestor had eyes too small to allow for successful aerial hawking of flying insects at night, but an auditory brain design sufficient to afford echolocation. Further, we find that among extant predatory bats (all of which use laryngeal echolocation), those with putatively less sophisticated biosonar have relatively larger eyes than do more sophisticated echolocators. We contend that signs of ancient trade-offs between vision and echolocation persist today, and that non-echolocating, phytophagous pteropodid bats may retain some of the necessary foundations for biosonar.

## Abstract (web summary)

Substantial evidence now supports the idea that the ancestral bat was a small, night flying predator capable of laryngeal echolocation. Here, the authors confirm this hypothesis using phylogenetic comparative analyses and further suggest an underlying tradeoff between echolocation and vision in both ancient and modern species and an association between sensory specialization and diet.

## Introduction

**Fig. 1:**
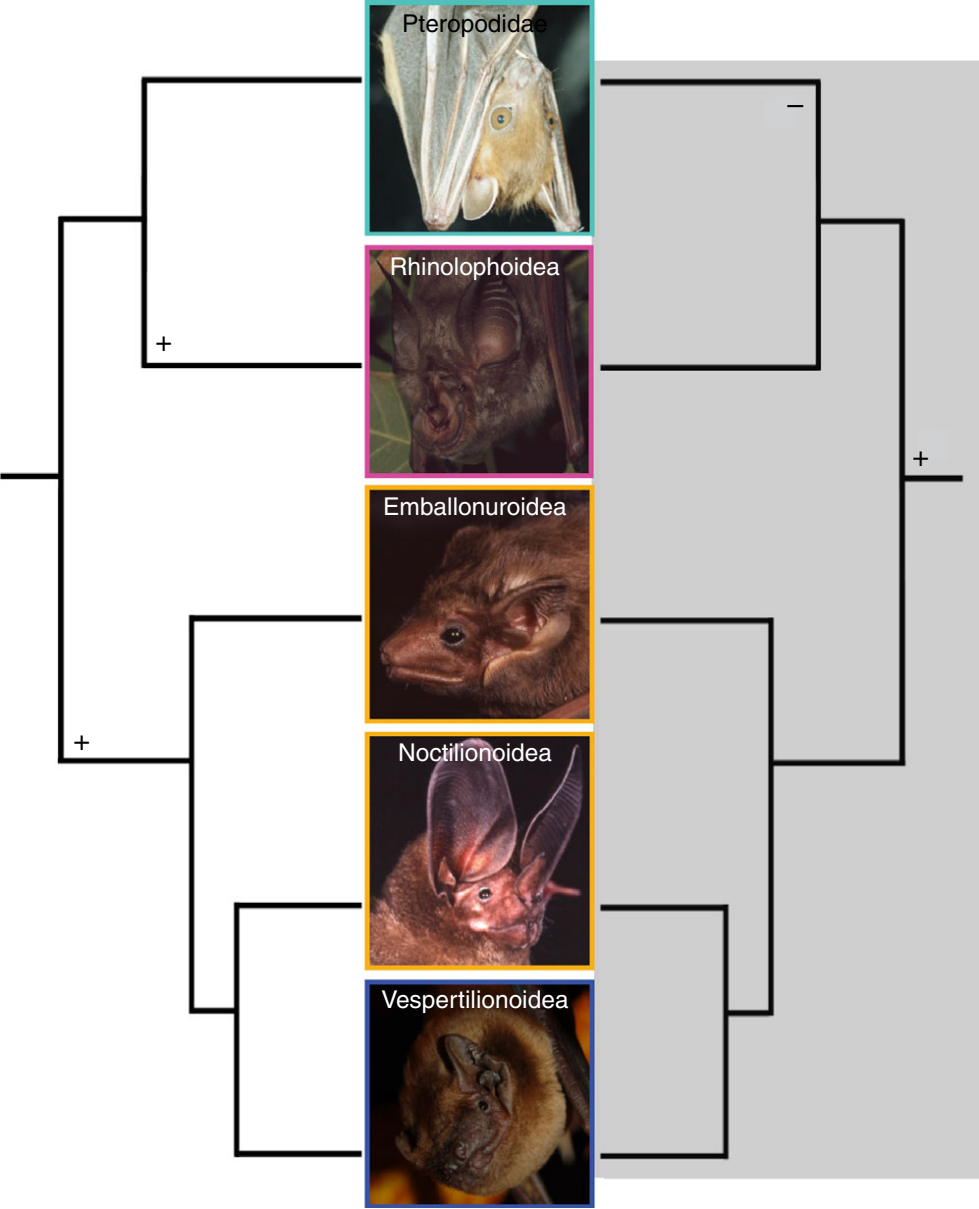
Two equally parsimonious hypotheses for the origination of laryngeal echolocation in bats. The unshaded side depicts the two origins hypothesis and predicts that laryngeal echolocation originated in the common ancestor to the Emballonuroidea, Noctilionoidea, and Vespertilionoidea and again in the Rhinolophoidea. The shaded side depicts the single origin hypothesis, which predicts laryngeal echolocation was present in the common ancestor of all bats and lost in the Pteropodidae. Middle column displays (top to bottom) five 30–35 g species from each of these major groups: *Cynopterus brachyotis* (non-echolocating, phytophagous), *Rhinolophus hildebrandti* (echolocating, predatory), *Taphozous melanopogon* (echolocating, predatory), *Tonatia evotis* (echolocating, predatory), *Nyctalus noctula* (echolocating, predatory). Please note that bats with constant frequency (CF), multi-harmonic frequency-modulated calls (MH) and fundamental harmonic frequency modulated calls (DH) (i.e., most energy in fundamental harmonic) are found in both suborders of bats. Photographs by Brock Fenton and Signe Brinkløv

Bats (Chiroptera) are the second largest order of mammals, comprising >1300 species and characterized by powered flight^1^. The vast majority of bats are strictly nocturnal^2^, with a few species also active around dawn and dusk^3,4^. Long before the discovery of echolocation, bats were once divided into “megabats” (members of the family Pteropodidae) and “microbats” (the remaining ~20 chiropteran families)^1,5–7^. Today these are anachronistic terms, and the pteropodids [(~200 vision-dependent species, none using laryngeal echolocation (LE)] are placed in Yinpterochiroptera (a.k.a. Pteropodiformes), which together with Yangochiroptera (a.k.a. Vespertilioniformes) comprise the two chiropteran suborders. Both suborders are otherwise comprised solely of laryngeal echolocators^6,7^. From a strict parsimony perspective, LE, if considered as a single trait, could therefore have evolved once in bats, and subsequently been lost in the pteropodids. Alternatively, LE could have evolved at least twice independently, once or more in Yangochiroptera, and once or more in Yinpterochiroptera, after the pteropodids diverged^6,8^ (Fig. 1). The sum of evidence, however, indicates (i) that the bats’ common ancestor was a predatory laryngeal echolocator and (ii) that the phytophagous pteropodids have lost most, but perhaps not all, hallmarks of this complex active sensory system^8–12^.

Since Donald Griffin’s discovery of echolocation^5,13^, the prevailing view has been that the first bats were small, nocturnal, insectivorous echolocators^6,14–16^. Specifically, bats are thought to have originated ≥64 mya^17,18^ to exploit the then unrealized foraging niche of small, night-flying insects, a resource most bats rely on today^5,12,15,19^. In darkness and dim light, nascent echolocation would have allowed these bats to pursue these then vulnerable insects^5,15^. Griffin^5^ too divided bats into those that use laryngeal echolocation (hereafter, LE bats) and those that do not (i.e., the pteropodids). Within LE bats, he identified three groups: (i) bats producing multi-harmonic (MH) calls, with little frequency modulation, (ii) bats producing downward-sweeping, frequency-modulated calls, with most energy in the fundamental harmonic, and (iii) bats producing long, constant frequency calls, with energy typically concentrated in the second harmonic^5^.

These four sensory divisions—non-laryngeal echolocators (hereafter, pteropodids or NLE bats), multi-harmonic echolocators (MH bats), frequency modulating, dominant harmonic echolocators (DH bats), and constant frequency echolocators (CF bats)—remain robust functional descriptors of biosonar diversity in bats^6,20^. Griffin^5^ and others since^6,12,14,21–24^ have suggested that MH calls most closely reflect bats’ ancestral condition. If so, pteropodids, DH, and CF bats would represent three derived sensory states^6,10,12^. While no pteropdodid is thought to be predatory or to be capable of LE, members of the genus *Rousettus* use biosonar based on tongue clicking for orientation^25,26^. This form of echolocation, while effective, falls short of the maximum detection distances and update rates observed in LE bats^19,25,26^. Among LE bats, it has been previously argued that CF and DH bats possess more advanced abilities for detecting and tracking flying prey than do MH bats^5,27–30^. Similarly, different visual abilities exist within today’s bats, with pteropodids possessing the most advanced visual systems, in some species including an optic chiasm^31^, and the LE vespertilionids and rhinolophids perhaps the least^32^. Interestingly, all rhinolophids use CF calls, while most vespertilionids use DH calls^12,20,29^. It is among the MH species that we find the LE bats capable of the greatest quantified visual resolution^32,33^ and even ultraviolet light sensitivity^34^.

Here, we use phylogenetic comparative methods to further test the hypothesis that the ancestral bat was a small, predatory echolocator, which produced MH biosonar signals^5,12,14^. Additionally, we test three hypotheses about the relationships between visual abilities and echolocation behavior in bats across their four sensory divisions, and with respect to diet and roosting behavior, relative to ancestral states (ASs). These three hypotheses reflect mechanistic explanations for the origination and evolution of LE in bats for pursuing flying insects, and predict auditory opportunity and visual constraint^5,35^. Specifically, we test predictions that the ancestral bat had (i) an auditory brain design capable of supporting early LE, but (ii) eyes of insufficient absolute size to allow insect tracking at night. We also test the predictions that today not only would pteropodids possess relatively larger eyes than LE bats but that among predatory bats (all of which use LE), (i) MH bats would have relatively larger eyes than DH and CF bats and (ii) short-wavelength-sensitive (SWS) opsin genes would remain functional in MH and DH bats, but have lost functionality in CF species^36^.

Using modern phylogenetic comparative methods and a recent bat molecular phylogeny, we find support for each of these four hypotheses. Specifically, our analyses unambiguously support the idea that the common ancestor of modern bats was a small, flying nocturnal predator capable of LE and that this complex sensory trait has regressed in the pteropodids. Further, our results suggest that this vocal–auditory solution was favored over vision due to pre-existing sensory opportunities and constraints and that these sensory trade-offs persist to different extents in bats today.

## Results

### Estimated states and traits of the ancestral bat

**Fig. 2:**
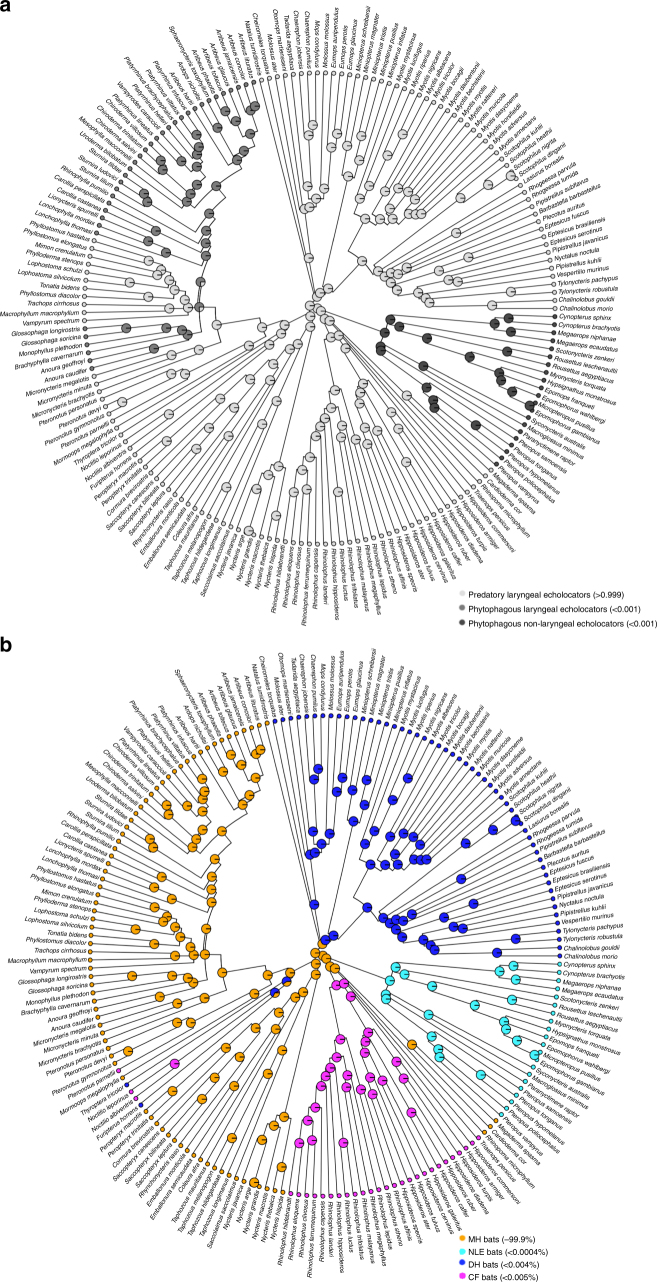
The ancestral state estimates of call types and foraging categories. **a** The echolocation signals of bat species (*N* = 183) were categorized as (i) constant frequency (CF), (ii) multi-harmonic calls (MH), (iii) frequency modulated calls dominated by the fundamental harmonic (DH), or non-laryngeal (NLE, i.e., pteropodids). Models of evolution were compared using AICc scores and the character states for ancestral call types were estimated under an equal rates model of evolution. These marginal ancestral states (i.e., the empirical Bayesian posterior probabilities) have been overlain on the phylogeny. We find support for a multi-harmonic ancestral call type (Bayesian posterior probabilities: CF: <0.001; MH: >0.999; DH: <0.001; MLE: <0.001). **b** Bats were also categorized as (i) predatory laryngeal echolocators (ALE), (ii) phytophagous laryngeal echolocators (PLE) and (iii) phytophagous non-laryngeal echolocators (PNLE). Models of evolution were compared using AICc scores and the character states for ancestral call types were estimated under an equal rates model of evolution. These marginal ancestral states have been overlain on the phylogeny. Our results suggest that the ancestral bat was a predatory laryngeal echolocator (Bayesian posterior probabilities: ALE: >0.999; PLE: <0.001; PNLE: <0.001)

We estimated Bayesian posterior probabilities for both foraging strategy and call type under an equal rates (EQ) model of evolution, as this model produced the lowest AICc scores for both categories (foraging category: EQ AICc = 45.43, symmetric transition (SYM) AICc = 48.51, all-rates-different (ARD) AICc = 53.35; call design categories: EQ (AICc) = 41.2, SYM = 44.17, ARD = 49.44). We found an additional support for the hypothesis that the ancestral bat was a predatory, echolocating bat (Bayesian posterior probabilities: animal-eating laryngeal echolocator >0.999, phytophagous laryngeal echolocator <0.001, phytophagous NLE <0.001; Fig. 2a). These same results support the idea that phytophagy has originated at least twice in the Chiroptera, once in the Pteropodidae and at least once in the Phyllostomidae (Fig. 2a). With respect to biosonar signal design, we found support for the hypothesis that the ancestral bat produced multiharmonic calls (Bayesian posterior probabilities: MH >0.999, constant frequency <0.001, fundamental harmonic frequency modulated <0.001; non-laryngeal echolocating: <0.001; Fig. 2b).

**Fig. 3:**
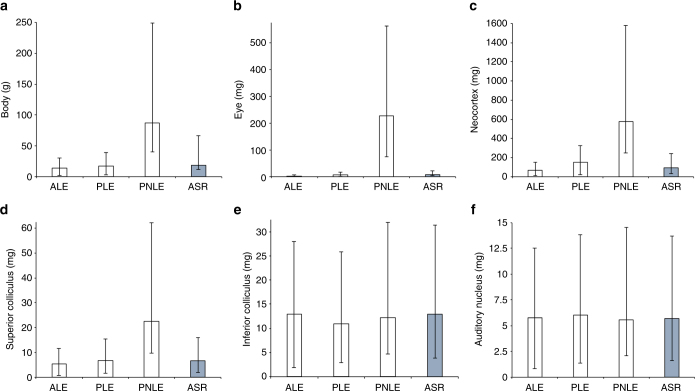
The ancestral states of bats versus modern foraging categories. The ancestral states (maximum likelihood estimate of the root node) of six continuous traits considered in this study are shown with 95% confidence intervals. The tree was re-rooted at each internal nodes and contrasts state at the root was computed each time. AS estimate at the root compared to extant foraging categories for: **a** body mass (*N* = 183), **b** eye mass (*N* = 183), **c** neocortex mass (*N* = 149), **d** superior colliculus mass (*N* = 84), **e** inferior colliculus mass (*N* = 84), and **f** auditory nucleus mass (*N* = 84). The ancestral state range of eye mass and non-auditory brain regions (**b-d**) suggest an increase in pteropodids, while those of the auditory regions (**e**, **f**) suggest a basic auditory brain design has been conserved in all bats. We found that the auditory regions (i.e., inferior colliculus, auditory nucleus) were the only brain regions that did not differ between the ancestral bat and today’s species (see also Supplementary Fig. 1), supporting the notion that the ancestral bat had an auditory brain sufficient for echolocation

We reconstructed ASs of body and brain mass. These reconstructions suggest that the ancestral bat was ~20 g, roughly the mean size of today’s laryngeal echolocating bats, and smaller than most extant pteropodid bats (Fig. 3; Supplementary Fig. 1), with a relative brain mass >20% smaller than that of extant pteropodid species (body mass: *N* = 183, root AS = 18.55 g, 95% confidence interval (CI) = 7.18 (lower limit), 47.91 (upper limit); brain mass: *N* = 183; AS = 428.33 mg, CI = 229.59, 799.08), confirming a previous report^37^. For comparison with AS reconstructions of auditory brain regions (see below) and for comparison with modern day bats, we also reconstructed the ASs of several non-auditory brain region masses associated with sensory information processing (neocortex: *N* = 149; AS = 94.15 mg, CI = 61.25, 144.71; hippocampus: *N* = 149; AS = 26.53 mg, CI = 18.24, 38.58; olfactory bulb: *N* = 149; AS = 9.02 mg, CI = 5.8, 14.05; superior colliculus: *N* = 84; AS = 6.66 mg, CI = 4.69, 9.45; Fig. 3; Supplementary Fig. 1).

### Phylogenetic signal

To estimate the degree to which phylogeny predicts the pattern of covariance among species, we used a recent molecular phylogenetic tree^38^ and Pagel’s lambda^39^. We found significant phylogenetic signal for all log-transformed variables (body mass: *λ* = 0.9787; log*L* = −210.37, *p* < 0.001; brain mass: *λ* = 0.82; log*L* = −143.59, *p* < 0.001; eye mass: *λ* = 0.95; log*L* = −351.73, *p* < 0.001; log*L* = −55.85, *p* < 0.001; neocortex: *λ* = 0.95; log*L* = −135.26, *p* < 0.001; hippocampus: *λ* = 0.92; log*L* = −187.85, *p* < 0.001; superior colliculus: *λ* = 1.0; log*L* = −72.65, *p* < 0.001; olfactory bulb: *λ* = 0.97; log*L* = −146.76, *p* < 0.001; inferior colliculus: *λ* = 0.87; log*L* = −72.79, *p* < 0.002; auditory nucleus: *λ* = 0.84; log*L* = −68.21, *p* < 0.001).

### Mass-residuals

We show (using phylogenetic generalized least-squares by restricted maximum likelihood) that brain and eye mass were positively correlated with body mass (brain: *b* = 0.654 ± 0.010; *t* = 64.917; *p* < 0.001; *R*^2^ = 0.877; eye: *b* = 0.745 ± 0.056; *t* = 13.197; *p* < 0.001; *R*^2^ = 0.705), as were brain regions (inferior colliculus: *b* = 0.566 ± 0.030; *t* = 18.673 *p* < 0.001, *R*^2^ = 0.295; auditory nucleus: *b* = 0.578 ± 0.000; *t* = 745,121.1, *p* < 0.001, *R*^2^ = 0.246; superior colliculus: *b* = 0.590 ± 0.021; *t* = 28.344, *p* < 0.001, *R*^2^ = 0.930; olfactory bulb: *b* = 0.726 ± 0.035; *t* = 20.895, *p* < 0.001, *R*^2^ = 0.800; hippocampus: *b* = 0.603 ± 0.022; *t* = 26.896, *p* < 0.001, *R*^2^ = 0.740; neocortex: *b* = 0.710 ± 0.000; *t* = 9,202,949, *p* < 0.001, *R*^2^ = 0.879). Thus, we generated phylogenetic residuals for log-transformed brain mass, eye size, and brain region masses on body mass, and tested for differences in these residuals across our three foraging strategies and four biosonar signal designs.

### Ancestral brain regions versus modern foraging categories

Phylogenetic analyses of variance (ANOVAs) indicate that pteropodid bats are significantly larger than animal-eating bats (*F* = 40.353, *p* = 0.03; Supplementary Table 1). We also found that absolute brain, neocortex, hippocampus, and olfactory bulb sizes are significantly larger in pteropodids than in animal-eating bats (brain: *F* = 70.763, *p* = 0.009; neocortex: *F* = 55.618, *p* = 0.006; hippocampus: *F* = 82.641, *p* = 0.001; olfactory bulb: *F* = 85.068, *p* = 0.001; Supplementary Table 1). AS reconstructions suggest that these structures have become larger in pteropodids, while the neocortex and olfactory bulb may have become smaller in animal-eating species (Fig. 3; Supplementary Fig. 1). We found that absolute superior colliculi are larger in pteropodid bats than in animal-eating bats (*F* = 26.937, *p* = 0.014; Supplementary Table 1) and larger in pteropodids compared to ancestral reconstructions, suggesting greater investment in visual tracking (Fig. 3; Supplementary Fig. 1). For each of these traits, the phytophagous phyllostomids fell somewhere between the pteropodids and animal-eating bats, and did not differ from either of these groups significantly (Supplementary Table 1).

We, like previous authors^40–42^, found that phytophagous species in general have relatively (i.e., phylogenetically informed mass-residuals) larger brains (*F* = 113.747, *p* = 0.001) than predatory bats. Also, like previous studies, we found that phytophagous species have relatively larger neocortices (*F* = 80.525, *p* = 0.002), hippocampi *F* = 126.534, *p* = 0.001), olfactory bulbs *F* = 129.473, *p* = 0.001) than do predatory species^41,43^. Additionally, we found that the phytophagous bats also had relatively larger superior colliculi (*F* = 35.649, *p* = 0.006) than animal-eaters (Supplementary Table 2).

### Ancestral brain regions versus modern echolocation categories

With respect to echolocation signal design, we found that bats that do not produce echolocation signals using their larynges (i.e., the pteropodids) had larger absolute brains, neocortices, hippocampi and olfactory bulbs than did CF and DH bats (brain: *F* = 49.96, *p* = 0.016, neocortex: *F* = 41.58, *p* = 0.016, hippocampus: *F* = 43.16, *p* = 0.017, olfactory bulb: *F* = 35.09, *p* = 0.025) and larger absolute superior colliculi than DH bats (brain: *F* = 21.36, *p* = 0.011) (see Supplementary Table 3).

In relative terms, we found that the pteropodids had larger relative brains, neocortices, olfactory bulbs than both CF and DH bats (brain: *F* = 84.54, *p* = 0.002, neocortex: *F* = 90.16, *p* = 0.001, olfactory bulb: *F* = 25.45, *p* = 0.016), larger relative hippocampi than DH bats (*F* = 35.82, *p* = 0.023), and larger relative superior colliculi than all laryngeal echolocating bats, regardless of call type (*F* = 44, *p* = 0.001). Other than with respect to relative superior colliculus size, MH bats fell between the pteropodids, on the one hand, and the CF and DH bats, on the other, for all other measures of relative brain and brain region size (see Supplementary Table 4).

### Ancient auditory brain versus modern foraging categories

With respect to relative auditory brain region size, we found the opposite trends to those above for the inferior colliculi and auditory nuclei. Specifically, pteropodid auditory brain regions were relatively smaller than those of laryngeal echolocating bats^41^ (inferior colliculus: *F* = 73.291, *p* = 0.001; auditory nucleus: *F* = 58.585, *p* = 0.001; Supplementary Table 2).

However, we found no differences between foraging categories with respect to the absolute masses of the auditory brain regions: absolute inferior colliculus size (*F* = 0.323, *p* = 0.946) and absolute auditory nucleus size (*F* = 0.043, *p* = 0.992), which remain similar across these three categories (Supplementary Table 1). Neither did we observe any differences in the sizes of auditory brain regions in modern bats relative to the common ancestor (inferior colliculus: *N* = 84; AS = 12.93 mg, CI = 9.09, 18.4; auditory nucleus: *N* = 84; AS = 5.7 mg, CI = 4.08, 7.97) (Fig. 3; Supplementary Fig. 1; Supplementary Table 1).

### Ancient auditory brain versus modern echolocation categories

With respect to biosonar signal design categories, we found that there were no significant differences in the absolute auditory regions of the non-laryngeal echolocating pteropodids and any of the laryngeal echolocating bats, regardless of signal design (inferior colliculus: *F* = 1.558, *p* = 0.826, auditory nucleus: *F* = 1.558, *p* = 0.817; Supplementary Table 3). In relative terms, we found that the pteropodids had smaller auditory regions than laryngeal echolocating bats, regardless of call type (inferior colliculus: *F* = 42.598, *p* = 0.001, auditory nucleus: *F* = 47.309, *p* = 0.001) (see Supplementary Table 4).

### Eye size in the ancestral bat versus in modern bats

We found that the Plasticine models best predicted eye diameters reported in the literature, and thus used these as proxies for eye diameter in our analyses (Plasticine model: *R*^2^ = 0.9, *p* < 0.001; eyelid length: *R*^2^ = 0.78, *p* < 0.001; ZB–IOD: *R*^2^ = 0.3, *p* < 0.002). Using these estimates, we reconstructed the AS of absolute eye size (*N* = 183; AS = 7.67 mg, CI = −20.23, 110.09; Fig. 3). This translates into an ancestral eye diameter of 3.13 mm. This is smaller than the smallest pteropodid eye found today (*Syconycteris australis* with a diameter of 5.03 mm) and similar in size to that of the largest extant phytophagous and predatory laryngeal echolocating bats (see Supplementary Data 1).

We also confirmed that pteropodids have absolutely larger eyes than do laryngeal echolocators (*F* = 149.248, *p* = 0.001; Supplementary Table 1), and compared to the AS estimate at the root node, this suggests a trend of increasing eye size in pteropodids and possible reduction in eye size in most extant laryngeal echolocating bats (Fig. 3; Supplementary Fig. 1). We also found that in relative terms, the non-laryngeal echolocating pteropodids had larger eyes than laryngeal echolocating bats, regardless of diet (*F* = 88.362, *p* < 0.001) (Supplementary Table 2) or call type (absolute: *F* = 136.18, *p* = 0.001; relative: *F* = 146.86, *p* = 0.001) (see Supplementary Tables 3 and 4).

**Fig. 4:**
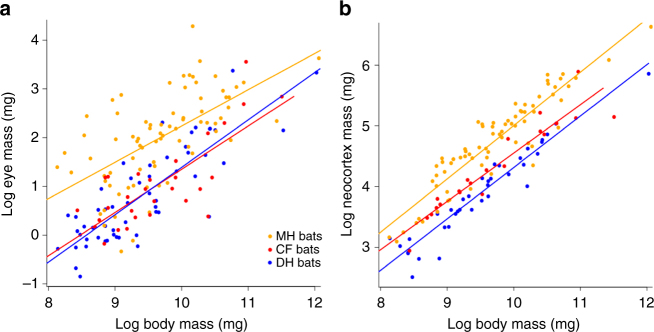
Phylogenetically informed linear regressions of eye mass and neocortex on body mass by call type. Phylogenetic generalized least square models showing the regressions of log-transformed **a** eye mass (*N* = 162) and **b** neocortex (*N* = 162) on body mass while accounting for the phylogenetic non-independence between data points. The calls of laryngeal echolocating bats were categorized as (i) constant frequency (CF), (ii) multi-harmonic calls (MH), (iii) frequency modulated calls dominated by the fundamental harmonic (DH) and are shown separately on the plots

To consider the relationship between visual investment and echolocation sophistication, without the confounding effects of diet, we then considered the relationships between all traits and echolocation call design in only predatory species (i.e., after removing all pteropodids and all phytophagous phyllostomids). That is, between predatory bats producing MH calls (AS), and CF and DH calls (both derived). We found no differences between MH, DH, and CF predatory bats with respect to absolute body, brain, brain region, nor eye mass (i.e., all traits considered; Supplementary Table 5). However, we found differences among these bats with respect to relative eye mass (*F* = 31.450, *p* = 0.048), neocortex mass (*F* = 41.499, *p* = 0.004), and superior colliculus mass (*F* = 14.256 *p* = 0.048) (Fig. 4; Supplementary Table 6).

Specifically, we found that relative eye size was significantly larger in MH bats than in DH bats (*p* = 0.05), and nearly so in MH versus CF bats (*p* = 0.08). We found no difference in relative eye size between DH and CF bats (*p* = 0.957). Similarly, we found that relative neocortex was larger in MH than in DH bats (*p* = 0.003), and nearly so with respect to relative superior colliculus mass (*p* = 0.063) (Fig. 4; Supplementary Table 6). Last, as predicted^36^, we found that the ancestral bat likely possessed functional SWS opsin genes (Bayesian posterior probabilities: functional SWS opsin gene: 0.971; non-functional SWS opsin gene: 0.029), and that among predatory bats, these genes are now non-functional in CF bats but remain functional in MH and DH bats (*p* = 0.002) (Supplementary Fig. 3).

Because the above result surprised us, we wanted to consider the potential influence of roosting preference (i.e., whether light environment might impact relative eye size). However, after categorizing predatory species as roosting either exclusively in caves/cavities or as also using exposed roosts (Supplementary Data 1), we found no differences in absolute (*F* = 0.676, *p* = 0.427) or relative (*F* = 0.412, *p* = 0.545) eye mass between the 3 call type groups. We also found no significant relationship between these two roost categories and ancestral (MH) versus derived (CF + DH) call types (*p* = 0.950). We did however find that the ancestral bat was likely to have roosted using exposed surfaces, rather than caves or cavities (Bayesian posterior probabilities: exposed roosts: 0.998, caves/cavities: 0.002).

**Fig. 5:**
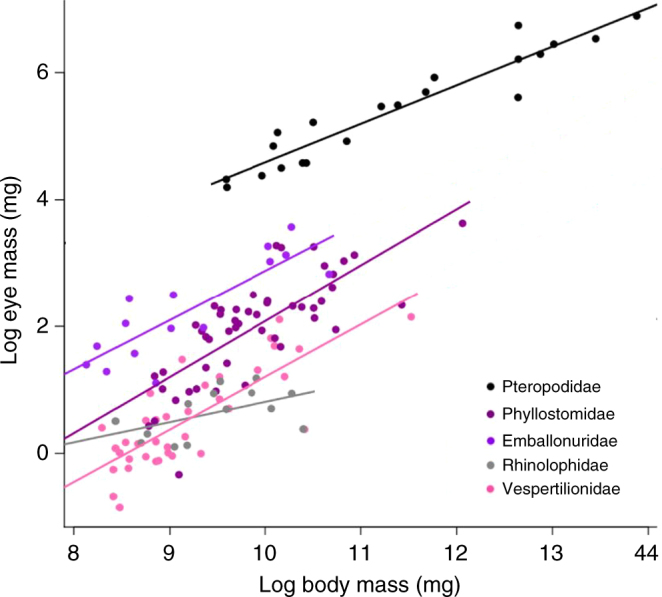
Phylogenetically informed linear regressions of brain and eye mass on body mass by family. Phylogenetic generalized least square models showing the regressions of log-transformed eye mass (*N* = 183) on body mass accounting for phylogenetic non-independence among data points. The 5 most speciose families of bats are shown separately on the plots. The pteropodids have the largest absolute eye size while the predatory emballonurids have the largest eyes among the laryngeal echolocating bats. The smallest eyes are found in the more advanced echolocating rhinolophids and vespertilionids, suggesting that there may be an echolocation-vision trade-off even among predatory species.

Figure 5 illustrates the phylogenetically informed linear regressions between eye and body mass in the five most speciose families of bats, and suggests the strictly predatory emballonurids (all MH bats) have the largest eyes among LE bats.

## Discussion

Our comparative analyses lend strong support to the already well-supported hypothesis that the common ancestor of bats was a small (~20 g), predatory, laryngeal echolocator^5,12,37^. Specifically, a bat that took flying insects on the wing at night^5^ and roosted externally, rather than deep in caves. Our results, thus, also support the conclusion that LE has been lost, rather than never gained, in the family Pteropodidae^8^ (Figs. 1–3). They also indicate that a switch to a phytophagous diet occurred at least twice in bats since their origin, once in the pteropodids (Yinpterochiroptera) and once or more within the laryngeal echolocating family Phyllostomidae (Yangochiroptera)^44^ (Fig. 2a).

We also confirm relative brain size is greater in phytophagous bats (i.e., the pteropodids and the laryngeal echolocating phytophagous phyllostomids) than today’s predatory bats^40–42,45^ (Supplementary Table 2; Supplementary Fig. 2), and compared to the common ancestor. This trend is largely due to enlargement of the olfactory bulb, hippocampus, and neocortex in phytophagous bats^41,43^ (Fig. 3; Supplementary Fig. 1). Further, our analyses demonstrate the ancestral bat had a relatively larger brain than some, but not all, extant predatory bat lineages, perhaps reconciling a current point of contention^37,46^ (Supplementary Fig. 2). Our results also support the hypothesis that this bat used multi-harmonic (MH) echolocation calls, and thus that constant-frequency (CF) and dominant-harmonic (DH) call designs are derived states^5,12,14,29^ (Fig. 2b).

Based on this hypothetical framework, we now consider three hypotheses about the evolution of bat echolocation. Specifically, we investigate what potential auditory opportunities and visual constraints may have characterized this common ancestor, and the past and present relationships between these senses in bats. A diet of night-flying insects is thought to have constrained the ancestral bat (and indeed most predatory bats today) to a small body size^47^. Based on our results, we contend that this nocturnal ancestor had an auditory system sufficient to afford echolocation, but eyes too small in absolute size—due to the constraints of small skull—to allow successful tracking of night-flying prey^35^. We also provide evidence that species-specific trade-offs between vision and sonar persist to this day.

To better understand vertebrate brain evolution, it is now established that we should consider not only brain and brain region size in relative terms, but in terms of absolute size. This is because absolute size better reflects processing power, neural investment, and information use^48^. Strikingly, although we confirmed that phytophagous species have relatively larger brains^40–42^ and non-auditory brain regions than today’s predatory bats^41,43^, and than the ancestral bat (Supplementary Table 2; Supplementary Fig. 2), we found that the ancestral bat’s auditory brain regions were of the same relative size as in extant predatory bats and had auditory regions roughly the same absolute size as those found in today’s LE bats (Fig. 3; Supplementary Table 1; Supplementary Fig. 1).

This outcome supports our hypothesis that the ancestral bat had sufficient auditory powers that could plausibly allow for a sonar solution in aid of detecting night flying insects. However, this bat would have almost certainly possessed a sonar system less sophisticated than those of today’s predatory bats. First, because ~65 million years of evolutionary refinement have since elapsed^5,12,19,22^. Second, sonar performance should also have improved because the night-active insects have since become better at evading pursuit and thus represent ~65 million years of selective pressures on most bats’ sonar systems to effectively track prey^15,19^. Indeed, while paleontological evidence suggests that the oldest known fossil bat, the insect-eating *Onychonycteris finneyi* (~52.5 mya), possessed the tympanal bone connection necessary for sonar target ranging^11^, the cochlea suggests frequency discrimination and upper frequency sensitivity inferior to that of most extant LE bats^49^.

Our results suggest to us that pteropodids have apparently maintained auditory brain regions of the same absolute size as the common ancestor and extant LE bats (Fig. 3; Supplementary Table 1; Supplementary Fig. 1). This lends support to the hypothesis that LE was lost, rather than never present, in this phytophagous lineage (Fig. 2a), as do several other lines of evidence. During prenatal cochlear development, pteropodids exhibit a rapid increase in cochlea size, similar to laryngeal echolocators and faster than other mammals^8^. They are also more sensitive to high frequency sounds than are similar-sized terrestrial mammals^9,26^. Indeed, the echolocation calls of most bats have peak frequencies between 20–60 kHz^12,15,50^, well within most pteropodids’ auditory limits^9,51^. Further, genetic vestiges suggest ancient biosonar abilities in the pteropodids^10^.

While LE is unknown in extant pteropodids–as is the case for echolocation of any kind in almost all ~200 pteropodid species–the biosonar-based orientation abilities of the tongue-clicking pteropodid, *Rousettus aegyptiacus*, have recently been recognized as being more sophisticated than previously thought^25^. Furthermore, more rudimentary echo-based orientation has now been experimentally supported in at least two other pteropodid genera, based on wing clicks potentially used in nature for finding suitable roosting places in dark caves^52,53^. Taken together, all of the above suggests that not only was LE lost, rather than never present, in the Pteropodidae, but that the foundations for chiropteran echolocation may not have regressed entirely and instead remain available to be built upon in this lineage. Indeed, this has, perhaps, happened several times already (see Fig. 3 in ref. ^53^).

Our results also demonstrate that echolocation may have originated first in the progenitors of bats, and only rarely in any vertebrate group thereafter^26,54^, not simply because they were pre-adapted for a sonar solution, but also because they were constrained by a small body^37,47^ (Fig. 3), and thus skull and orbit size^35^, from instead realizing a vision-based solution. That is, while our AS reconstruction indicates that the ancestral bat had relatively and absolutely larger eyes (~3 mm diameter) than most extant LE bats (Fig. 3; Supplementary Fig. 2; Supplementary Data 1), these same results reveal that their eyes were both relatively and absolutely smaller than those of all extant pteropodid species, including those pteropodid species smaller in body size than the ancestral bat (i.e., all extant pteropodid species have eyes >5 mm diameter, while the smallest species weigh ~15 g; Fig. 3; Supplementary Figs. 1, 2). As we outline below, vertebrate eyes of the size estimated for the ancestral bat would be, then and now, too small to allow for the successful aerial pursuit of even undefended flying insects at night.

For two closely related vertebrates of similar size, one nocturnal and the other diurnal, relatively larger eyes in the former is the norm and indicative of greater investment in vision to be able to acquire enough light to see adequately at night^55,56^. For example, crepuscular aerial insectivorous birds not only have disproportionately larger eyes but also have relatively larger skulls than otherwise similarly sized diurnal aerial insectivorous birds^55,57^, and have average body weights of ~50 g or more^47^. Thus, we suggest the ancestral bats’ skull may have been too small to afford eyes large enough to allow sufficient sensitivity and resolution to guide and control flight at low light intensities and successfully track and capture flying insects. Under this scenario, the reduction in relative eye size in extant LE bats as compared to ancestral bat would reflect a greater reliance on more sophisticated echolocation over evolutionary time and a reduced reliance on vision (Fig. 3, Supplementary Fig. 1). The loss of a functional SWS opsin gene in CF bats (Supplementary Table 7; Supplementary Fig. 3), likely resulting in monochromatic rather than dichromatic vision in these sophisticated echolocators^36^, supports the plausibility of this viewpoint.

Conversely, the sensory divergence of the pteropodids away from early LE and towards a primarily vision-based solution reflects a transition from an insect to plant-based diet. This shift to relatively larger, energy-rich, stationary food times would have allowed for larger bodies, skulls, and eyes, and selected for larger brain regions associated with vision, olfaction, and spatial memory^45^. LE likely regressed due to physiological cost and lack of stabilizing selection, given the reduced benefit of sonar for locating stationary ripe fruit and flowers relative to detecting and tracking small moving insects. Notably, almost no phytophagous bat is known to use derived (i.e., CF or DH) echolocation calls. The pteropodid *Rousettus aegyptiacus* is a tongue-clicking echolocator, while all phytophagous phyllostomids, but one^58^, use MH call designs (Supplementary Data 1).

Among predatory bats, all of which laryngeally echolocate, we found that those that produce strictly MH calls had relatively larger eyes than did DH and CF bats (Fig. 4, Supplementary Table 6). Our results and the conclusions of researchers before us^5,12,14,21^ indicate that MH calls most closely resemble those of the ancestral bat (Fig. 2b) and are closest in structure to those of non-echolocating terrestrial mammals^12,14^. Our results therefore suggest that although absolute and relative eye size has decreased in all extant lineages of predatory bats as compared to the common ancestor (Fig. 3, Supplementary Fig. 1, Supplementary Fig. 2), relative eye size has decreased least in MH bats and most in DH and CF bats. Our analyses suggest that this difference is not accounted for by roost preference, and suggest that the exclusively predatory emballonurids have eyes at least as large as those of phyllostomids (Fig. 5).

While relatively and absolutely larger eyes in MH bats suggest better night vision, DH and CF call designs are not only derived but may be superior for detecting and tracking flying prey. DH bats (and to a lesser extent, CF bats) can adjust their sonar beam shape to suit habitat and task (reviewed in ref. ^19^), while MH bats apparently cannot^28,30^. Additionally, only CF bats, and perhaps some DH bats, use acoustic glints resulting from echoes from insects’ flapping wing to detect targets in cluttered habitat (reviewed in ref. ^29^). With respect to production, DH and CF call designs require laryngeal specializations for harmonic suppression, steep downward frequency sweeps and, in the case of CF bats, constant frequency components^59^. Further, the cochlea of DH and CF bats are more specialized relative to non-echolocating mammals than are those of MH bats^60–62^. Interestingly, the only predatory bat known to “shut off” echolocation while hunting under bright moonlight is the MH bat, *Macrotus californicus*^63^. Thus, a trade-off between echolocation and vision in bats apparently endures to this day, and is not accounted for only by diet, but extends to bats that continue to hunt flying insects on the wing under the cover of night.

## Methods

### Species categorization and brain data

Bat species were classified according to two systems. First, as (i) a pteropodid species, (ii) a phytophagous laryngeal echolocating (LE) species (roughly two-thirds of extant phyllostomid species), or (iii) as animal-eating LE bats (all remaining species, representing all families except the Pteropodidae). Diet and foraging strategies were assigned based on behavioral observations from the literature^43,44,64–66^. That is, we did not further subdivide predatory bats into gleaners and trawlers because all predatory bats are apparently able to take prey on the wing^15,19,44,67^, but those species that have been reported to also glean and trawl prey may reflect observation and reporting biases^44^.

We also categorized each bat species to one of the four sensory categories put forth by Griffin^5^: (i) bats that do not use LE (i.e., pteropodids), (ii) LE bats that only produce multi-harmonic calls (MH bats), (iii) LE bats that can produce steeply downward sweeping calls with most energy in the fundamental harmonic (DH bats), and (iv) LE bats which produce constant frequency call designs (CF bats)^5,6,12,21^. We also categorized bats as roosting internally or under exposed conditions. For the list of species and categories, see Supplementary Data 1. Last, for those bat species in the phylogeny^38^ for which reliable genetic visual pigment data exist, we categorized species as having either functional or non-functional short-wavelength sensitive (SWS) opsin genes^36,68–71^ (see Supplementary Table 7).

The absolute and relative sizes of the brain and brain regions reflect cognitive and spatial memory performance and reflect the degree to which different sensory modalities are relied upon to acquire environmental information. We compared total brain mass and six brain regions among bats with different echolocating abilities, foraging strategies, and diets: the neocortex, hippocampus, olfactory bulb, superior colliculus, inferior colliculus, and auditory nucleus. The superior colliculus and olfactory bulb are primarily involved in tracking visual and processing odor stimuli, respectively^72^. The inferior colliculus and auditory nucleus are primarily devoted to processing auditory information^37,72^. The hippocampus plays an important role in memory and spatial information processing and the neocortex in higher order cognition and complex stimuli perception^72^. Mass, brain and brain region masses were taken from ref. ^72^.

### Phylogenetic signal

Closely related species tend to be more similar to one another than to those more distantly related, thus species data are not statistically independent^73^. We used phylogenetic comparative methods to control for this non-independence. We estimated the degree to which phylogeny predicts the pattern of covariance among species with Pagel’s lambda^39^ and the Shi and Rabosky^38^ tree. All subsequent analyses were phylogenetically informed.

### Continuous and categorical AS reconstruction

We used *phytools* (v. 0.5–38) to reconstruct ASs^74^ for all log-transformed continuous variables, which we then anti-logged. The confidence intervals of the ancestral estimates for each variable were then compared to species-level modern categories (Fig. 1). We also used AICc scores to determine the most appropriate model of rate evolution and with *phytools* (v. 0.5–38), estimated the scaled likelihoods of each AS^74^ at the root node for our three foraging categories, four call type categories, two roost categories and for the functionality of the SWS opsin gene. The probabilities of these ancestral character estimates have been overlain on the phylogenies in Fig. 2 and Supplementary Fig. 3.

### Eye size estimation

To estimate eye size without sacrificing bats, eyes were modeled for those species that (i) were found in ref. ^72^, (ii) occurred in the recent comprehensive molecular phylogeny of Shi and Rabosky^38^, and (iii) for which the Royal Ontario Museum (ROM, Toronto, Canada) or the Natural History Museum of Denmark (Copenhagen) had at least one intact adult skull. This resulted in 183 species (name-matched using the taxonomy and species binomials found in Wilson and Reeder^75^ representing 18 of 21 chiropteran families). Plasticine balls were made by hand to comfortably fit into the orbit of each skull, near the optic nerve foramen (using at least one male and one female whenever possible), as described and validated by Brooke and colleagues^55^. Ball diameter was measured using digital calipers and used as a proxy of species-specific eye diameter to estimate eye mass^55^. We further confirmed the validity of this non-lethal means of eye size estimation using two other methods (see below), and compared all estimates with fresh eye diameters from the literature.

First, using the same skulls, we took photographs (using a Nikon D40x digital SLR camera) of their dorsal surface. The maximum zygomatic breadth (ZB) and the least interorbital breadth (IOD) were measured and the difference between these measures was used as an alternative proxy of eye size. Second, we photographed the eyes of intact alcohol-preserved specimens at the ROM (150 of 183 species). Whenever possible, at least one male and one female were used. The horizontal palpebral aperture was used as a proxy for eye length, measured as the distance between the medial and lateral canthi. We exported all photos to Image J v. 1.49 (National Institutes of Health, USA) and took measurements three times for each specimen to obtain a mean value for each species, from which we estimated diameter. Last, we took the axial lengths of fresh eyes for 33 species from the literature^32^ and compared to the three potential proxies for eye size (i.e., Plasticine models, the difference between the ZB and IOD, and eyelid lengths from wet specimens). For eye and skull measurements, specimen numbers, and museum collections, see Supplementary Table 8.

### Phylogenetically informed comparisons among groups

To test for differences in log-transformed body, brain, and eye masses across diets, foraging strategies, and echolocation ability, we carried out phylogenetic ANOVAs^74^ (1000 iterations), using a pruned version of the most comprehensive molecular phylogeny currently available for bats^38^. To test for differences among groups, we conducted post hoc comparisons of means. The *p*-values for these comparisons were obtained via phylogenetic simulation and adjusted using the Holm–Bonferroni correction to account for multiple testing^76^. The relationships between total brain mass, eye size, and mass were modeled using phylogenetic generalized least-squares by Restricted Maximum Likelihood^77^ with R-package *ape*^78^.

Brain and eye mass were positively correlated with body mass. Thus, we generated phylogenetic residuals for log-transformed brain mass, eye mass, and brain region masses on body mass, and tested for differences in these residuals across our four call type categories and our three foraging categories. Summaries for the results are provided in-text and in Supplementary Tables 1–6. We also used Pagel’s binary character correlation test to explored whether there were significant correlations between where bats roost (i.e., internally, externally) and (i) absolute and relative eye size or (ii) echolocation call types^74^. Further, using the same test, we tested the prediction that among predatory bats, there would be a significant correlation between CF echolocation and the loss of functionality in SWS opsin genes. Last, we plotted regressions of log transformed eye mass on body mass in the five most species rich bat families, while accounting for the phylogenetic non-independence among species.

### Data availability

All data generated or analyzed during this study are included in this published article (and its supplementary information files).

## Electronic supplementary material

Supplementary Information

Description of Additional Supplementary Files

Supplementary Data 1

## References

[1] Fenton MB, Simmons NB (2015). *Bats: a World of Science and Mystery*.

[2] Maor R, Dayan T, Ferguson-Gow H, Jones KE (2017). Temporal niche expansion in mammals from a nocturnal ancestor after dinosaur extinction. Nat. Ecol. Evol..

[3] Moore NW (1975). The diurnal flight of the Azorean bat (*Nyctalus azoreum*) and the avifauna of the Azores. J. Zool..

[4] Russo D, Cistrone L, Garonna AP, Jones G (2009). The early bat catches the fly: daylight foraging in soprano pipistrelles. Mamm. Biol..

[5] Griffin, D. R. *Listening in the Dark* (Yale University Press, New Haven, 1958).

[6] Jones G, Teeling EC (2006). The evolution of echolocation in bats. Trends Ecol. Evol..

[7] Fenton MB, Ratcliffe JM (2010). Bats. Curr. Biol..

[8] Wang Z (2017). Prenatal development supports a single origin of laryngeal echolocation in bats. Nat. Ecol. Evol..

[9] Calford MB, McNally KI (1987). Hearing in flying foxes (Chiroptera: Pteropodidae). Aust. Mammal..

[10] Teeling EC (2009). Hear, hear: the convergent evolution of echolocation in bats?. Trends Ecol. Evol..

[11] Veselka N (2010). *A bony conn*ection signals laryngeal echolocation in bats. Nature.

[12] Collen, A. *The evolution of echolocation in bats: a comparative approach*. (University College London, 462 Doctoral dissertation, 2012).

[13] Griffin DR (1944). Echolocation by blind men, bats and radar. Science.

[14] Simmons JA, Stein RA (1980). Acoustic imaging in bat sonar: echolocation signals and the evolution of echolocation. J. Comp. Physiol..

[15] ter Hofstede HM, Ratcliffe JM (2016). Evolutionary escalation: the bat-moth arms race. J. Exp. Biol..

[16] Fenton MB, Ratcliffe JM (2017). Bats united by cochlear development. Nat. Ecol. Evol..

[17] Teeling EC (2005). A molecular phylogeny for bats illuminates biogeography and the fossil record. Science.

[18] Bininda-Emonds ORP (2007). The delayed rise of present-day mammals. Nature.

[19] Ratcliffe JM, Elemans CPH, Jakobsen L, Surlykke A (2013). How the bat got its buzz. Biol. Lett..

[20] Schnitzler HU, Kalko EKV (2001). Echolocation by insect-eating bats. Bioscience.

[21] Neuweiler G (2003). 2003. Evolutionary aspects of bat echolocation. J. Comp. Physiol. A.

[22] Schnitzler, H.-U., Kalko, E. K. V. & Denzinger, A. in *Echolocation in Bats and Dolphins* (eds Thomas, J. A., Moss, C. F., & Vater, M.) 331–338 (University of Chicago Press, Chicago, 2004).

[23] Ratcliffe JM, Raghuram H, Marimuthu G, Fullard JH, Fenton MB (2005). Hunting in unfamiliar space: echolocation in the Indian false vampire bat, *Megaderma lyra*, when gleaning prey. Behav. Ecol. Sociobiol..

[24] Ratcliffe JM, Jakobsen L, Kalko EKV, Surlykke A (2011). Frequency alternation and an offbeat rhythm indicate foraging behavior in the echolocating bat. Saccopteryx bilineata. J. Comp. Physiol. A..

[25] Yovel Y, Geva-Sagiv M, Ulanovsky N (2011). Click-based echolocation in bats: not so primitive after all. J. Comp. Physiol. A.

[26] Brinkløv S, Fenton MB, Ratcliffe JM (2013). Echolocation in Oilbirds and swiftlets. Front. Physiol..

[27] Jakobsen L, Surlykke A (2010). Vespertilionid bats control the width of their biosonar sound beam dynamically during prey pursuit. Proc. Natl. Acad. Sci..

[28] Brinkløv S, Jakobsen L, Ratcliffe JM, Kalko EKV, Surlykke A (2011). Echolocation call intensity and directionality in flying short-tailed fruit bats, *Carollia perspicillata* (Phyllostomidae). J. Acoust. Soc. Am..

[29] Fenton MB, Faure PA, Ratcliffe JM (2012). Evolution of high duty cycle echolocation in bats. J. Exp. Biol..

[30] Jakobsen L, Olsen MN, Surlykke A (2015). Dynamics of the echolocation beam during prey pursuit in aerial hawking bats. Proc. Natl. Acad. Sci..

[31] Pettigrew JD (1986). Flying primates? Megabats have the advanced pathway from eye to midbrain. Science.

[32] Eklöf J. *Vision in echolocating bats.* (Göteborg University, Doctoral dissertation, 2003).

[33] Bell GP, Fenton MB (1986). Visual acuity, sensitivity and binocularity in a gleaning insectivorous bat, *Macrotus californicus* (Chiroptera: Phyllostomidae). Anim. Behav..

[34] Winter Y, López J, Von Helversen O (2003). Ultraviolet vision in a bat. Nature.

[35] Speakman J (2008). A first for bats. Nature.

[36] Zhao H (2009). The evolution of color vision in nocturnal mammals. Proc. Natl. Acad. Sci. USA.

[37] Safi K, Seid MA, Dechmann DKN (2005). Bigger is not always better: when brains get smaller. Biol. Lett..

[38] Shi JJ, Rabosky DL (2015). Speciation dynamics during the global radiation of extant bats. Evolution.

[39] Pagel M (1999). Inferring the historical patterns of biological evolution. Nature.

[40] Eisenberg JF, Wilson DE (1978). Relative brain size and feeding strategies in the Chiroptera. Evolution.

[41] Hutcheon JM, Kirsch JW, Garland T (2002). A comparative analysis of brain size in relation to foraging ecology and phylogeny in the Chiroptera. Brain Behav. Evol..

[42] Jones KE, MacLarnon AM (2004). Affording larger brains: testing hypotheses of mammalian brain evolution on bats. Am. Nat..

[43] Safi K, Dechmann DKN (2005). Adaptation of brain regions to habitat complexity: a comparative analysis in bats (Chiroptera). Proc. Roy. Soc. Lond. B.

[44] Dechmann DKN, Safi K (2009). Comparative studies of brain evolution: a critical insight from the Chiroptera. Biol. Rev..

[45] Ratcliffe JM (2009). Neuroecology and diet selection in phyllostomid bats. Behav. Proc..

[46] Yao L (2012). Evolutionary change in the brain size of bats. Brain Behav. Evol..

[47] Barclay RM, Brigham RM (1991). Prey detection, dietary niche breadth, and body size in bats: why are aerial insectivorous bats so small?. Am. Nat..

[48] Deaner RO, Isler K, Burkart J, van Schaik CP (2007). Overall brain size, and not encephalization quotient, best predicts cognitive ability across non-human primates. Brain Behav. Evol..

[49] Simmons NB, Seymour KL, Habersetzer J, Gunnell GF (2008). Primitive early Eocene bat from Wyoming and the evolution of flight and echolocation. Nature.

[50] Fullard, J. H. in *Comparative Hearing: Insects* (eds Hoy, R. R., Popper, A. N. & Fay, R. R.) 279–326 (Springer, New York, NY, 1998).

[51] Heffner RS, Koay G, Heffner HE (2006). Hearing in large (*Eidolon helvum*) and small (*Cynopterus brachyotis*) non-echolocating fruit bats. Hear. Res..

[52] Gould EG (1988). Wing-clapping sounds of *Eonycteris spelaea* (Pteropodidae) in Malaysia. J. Mammal..

[53] Boonman A, Bumrungsri A, Yovel Y (2014). Nonecholocating fruit bats produce biosonar clicks with their wings. Curr. Biol..

[54] Thomas, J. A., Moss, C. F., & Vater, M. *Echolocation in Bats and Dolphins* (University of Chicago Press, Chicago, 2004).

[55] Brooke M, de L, Hanley S, Laughlin SB (1999). The scaling of eye size with body mass in birds. Proc. R. Soc. Lond. B.

[56] Cronin, T., Johnsen, S., Marshall, N. J. & Warrant, E. J. *Visual Ecology* (Princeton University Press, New Haven, 2014).

[57] Hall MI, Ross CF (2007). Eye shape and activity pattern in birds. J. Zool..

[58] Mora EC, Macías S (2007). Echolocation calls of Poey’s flower bat (*Phyllonycteris poeyi*) unlike those of other phyllostomids. Naturwissenschaften.

[59] Metzner, W. & Schuller, G. in *Handbook of Mammalian Vocalization: An Integrative Neuroscience Approach* (ed. Brudzynski, S. M.) 403–415 (Academic Press, Amsterdam, 2010).

[60] Pye A (1966). The structure of the cochlea in chiroptera. I. Microchiroptera: Emballonuroidea and Rhinolophoidea. J. Morphol..

[61] Pye A (1966). The structure of the cochlea in Chiroptera. II. The Megachiroptera and Vespertilionoidea of the Microchiroptera. J. Morphol..

[62] Pye A (1967). The structure of the cochlea in chiroptera III. Microchiroptera: Phyllostomatoidea. J. Morphol..

[63] Bell GP (1985). The sensory basis for prey selection by the Californian leaf-nosed bat, *Macrotus californicus*. Behav. Ecol. Sociobiol..

[64] Norberg UM, Rayner JMV (1987). Ecological morphology and flight in bats (Mammalia; Chiroptera): wing adaptations, flight performance, foraging strategy and echolocation. Philos. Trans. R Soc. Lond. B Biol. Sci..

[65] Findley, J. *Bats: A Community Perspective* (Cambridge University Press, Cambridge, 1993).

[66] Nowak, R. *Walker’s Bats of the World* (Johns Hopkins University Press, Baltimore, 1994).

[67] Ratcliffe JM, Fenton MB, Shettleworth SJ (2006). Behavioral flexibility positively correlated with relative brain volume in predatory bats. Brain Behav. Evol..

[68] Müller B, Goodman SM, Peichl L (2007). Cone photoreceptor diversity in the retinas of fruit bats (Megachiroptera). Brain Behav. Evol..

[69] Müller B (2009). Bat eyes have ultraviolet-sensitive cone photoreceptors. PLoS One.

[70] Zhao H, Xu D, Zhou Y, Flanders J, Zhang S (2009). Evolution of opsin genes reveals a functional role of vision in the echolocating little brown bat (*Myotis lucifugus*). Biochem. Syst. Ecol..

[71] Melin AD, Danosi CF, McCracken GF, Dominy NJ (2014). Dichromatic vision in a fruit bat with diurnal proclivities: the Samoan flying fox (*Pteropus samoensis*). J. Comp. Physiol. A.

[72] Baron G, Stephan H, Frahm HD (1996). *Comparative Neurobiology in Chiroptera*.

[73] Felsenstein J (1985). Phylogenies and the comparative method. Am. Nat..

[74] Revell LJ (2012). Phytools: an R package for phylogenetic comparative biology (and other things). Methods Ecol. Evol..

[75] Wilson, D. E. & Reeder, D. M. *Mammal Species of the World: A Taxonomic and Geographic Reference* 3rd edition (Johns Hopkins University Press, Baltimore, 2005).

[76] Holm S (1979). A simple sequentially rejective multiple test procedure. Scand. J. Stat..

[77] Martins EP, Hansen TF (1997). Phylogenies and the comparative method: a general approach to incorporating phylogenetic information into the analysis of interspecific data. Am. Nat..

[78] Paradis E, Claude J, Strimmer K (2004). APE: analyses of phylogenetics and evolution in R language. Bioinformatics.

