# Supplementary material for: Auditory opportunity and visual constraint enabled the evolution of echolocation in bats
Source: Nat Commun. 2018 Jan 8;9:98. doi: 10.1038/s41467-017-02532-x (PMC5758785; doi:10.1038/s41467-017-02532-x)
Supplement: Supplementary file 1 — Supplementary Information [file 41467_2017_2532_MOESM1_ESM.pdf]

Supplementary Figure 1. Phylogenetic reconstructions of absolute continuous traits. Estimates of the maximum likelihood (ML) ancestral states for absolute (a) body, (b) eye, (c) brain, and (d-i) brain regions. Masses were generated and mapped onto the tree by estimating states at internal nodes using ML and interpolating the states along each edge. The phylogeny and variation in the absolute trait values were combined to visualize the increase and decrease of these traits in different lineages over evolutionary time. The color gradient indicates trait size, as mapped onto the phylogeny (families indicated along right side).

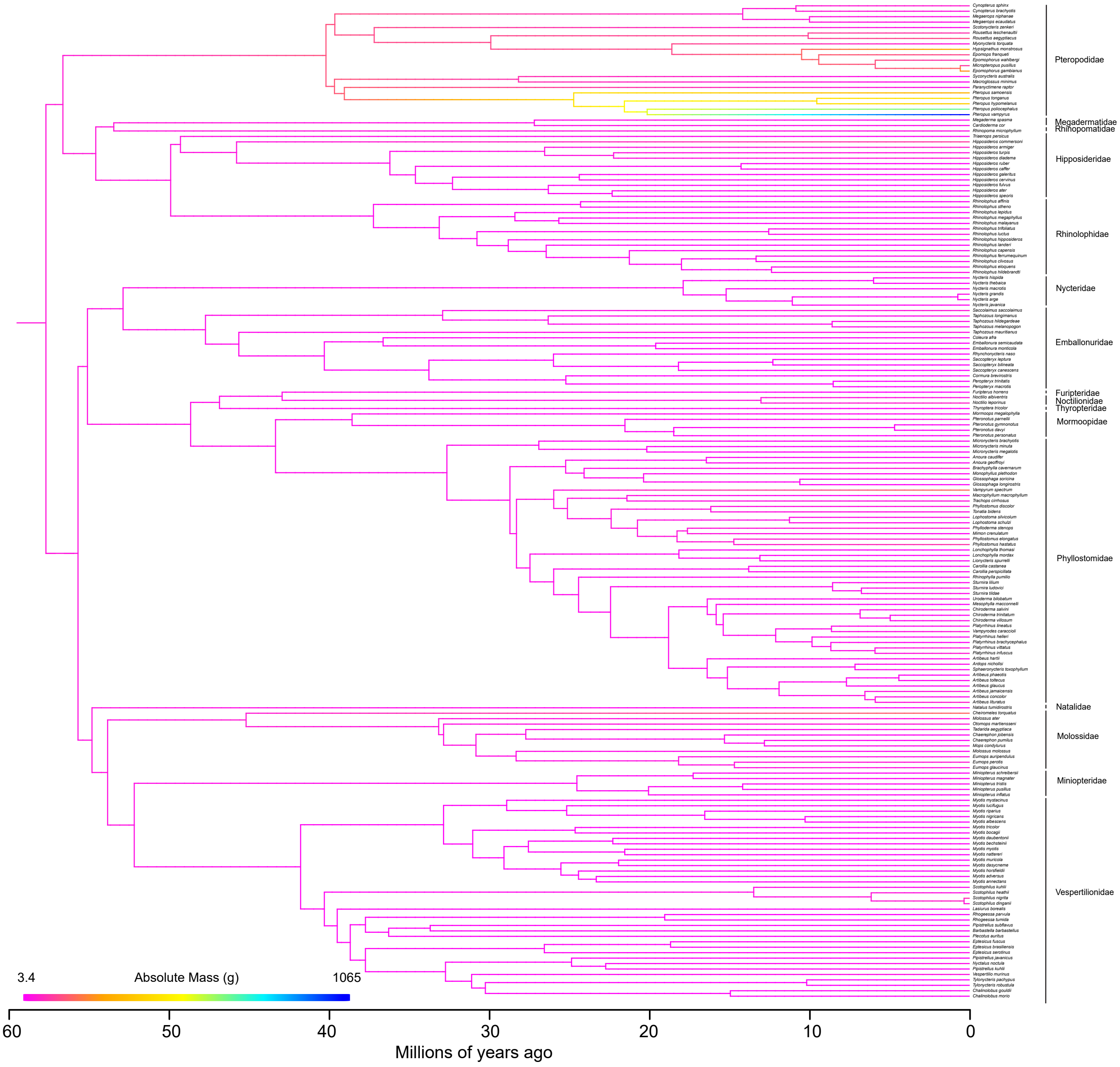

Supplementary Figure 1a.

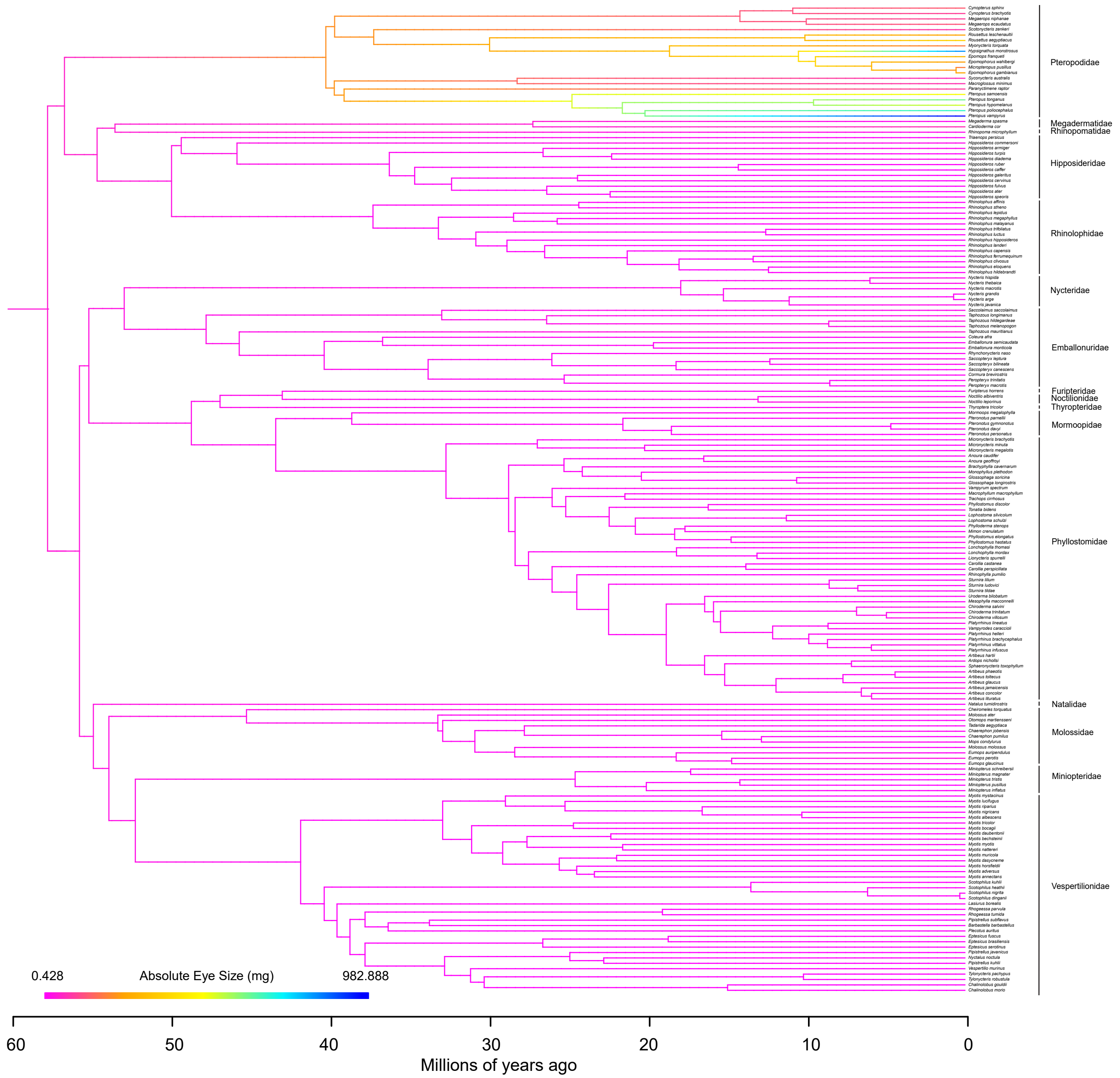

Supplementary Figure 1b.

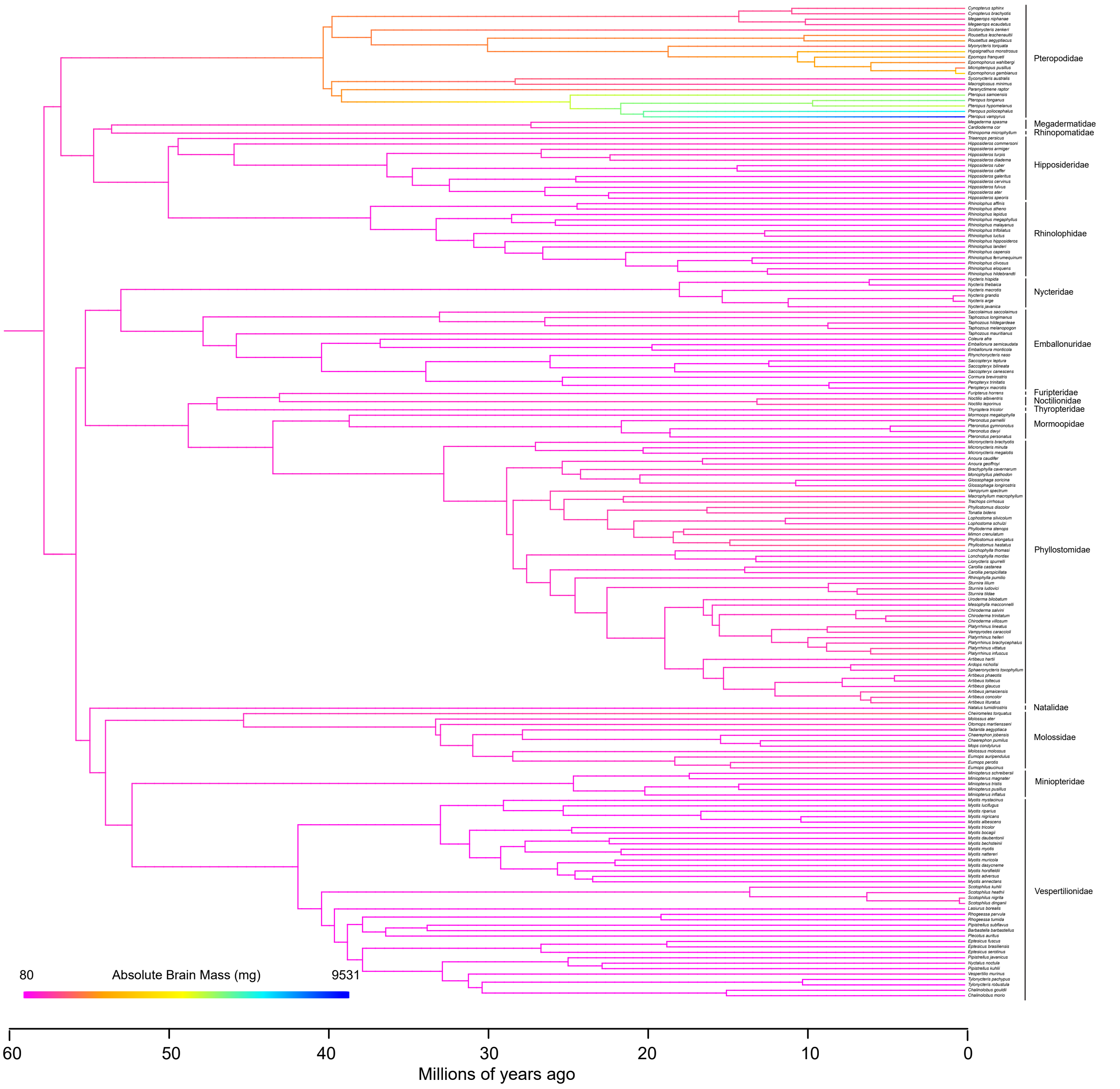

Supplementary Figure 1c.

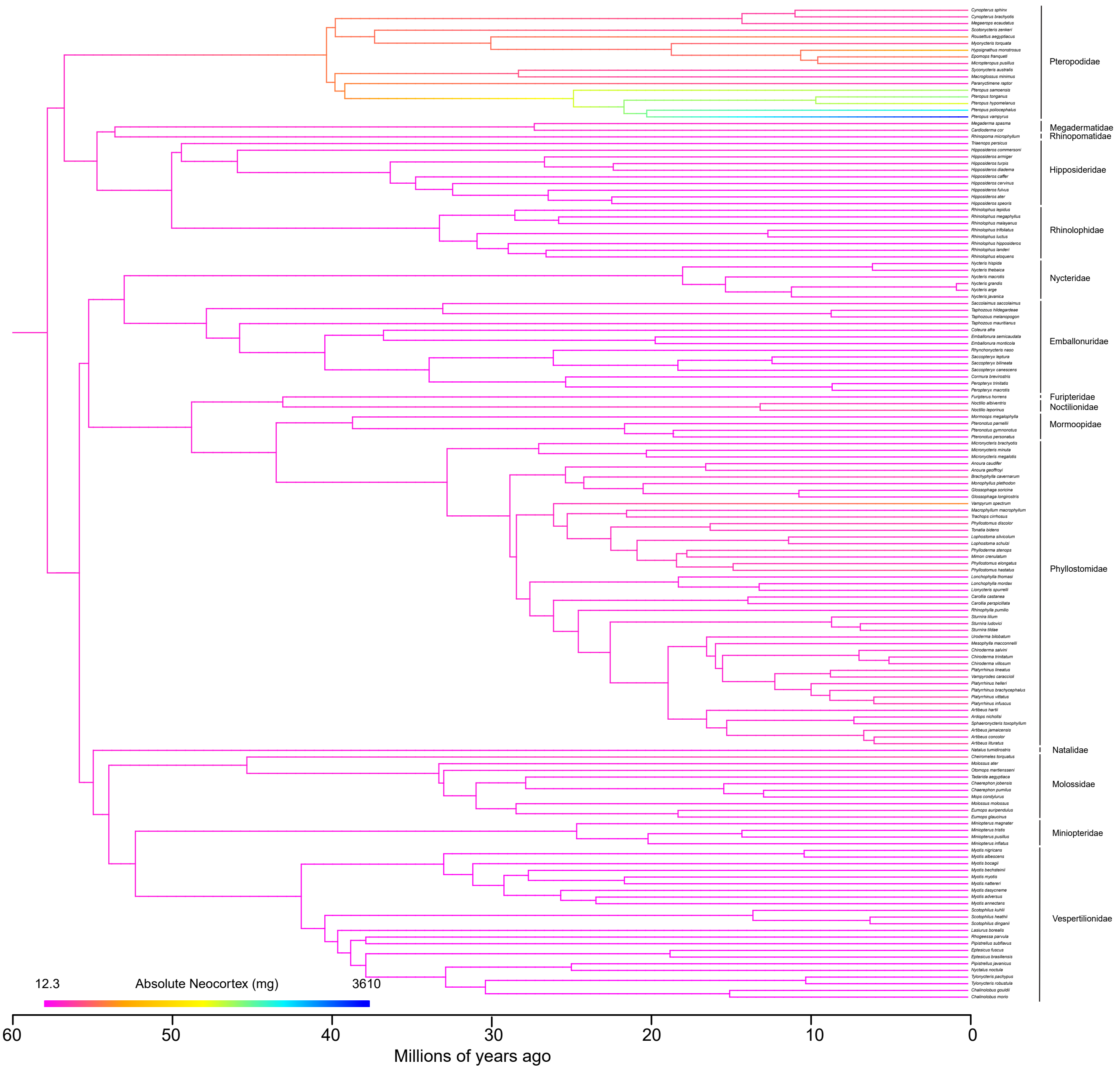

Supplementary Figure 1d.

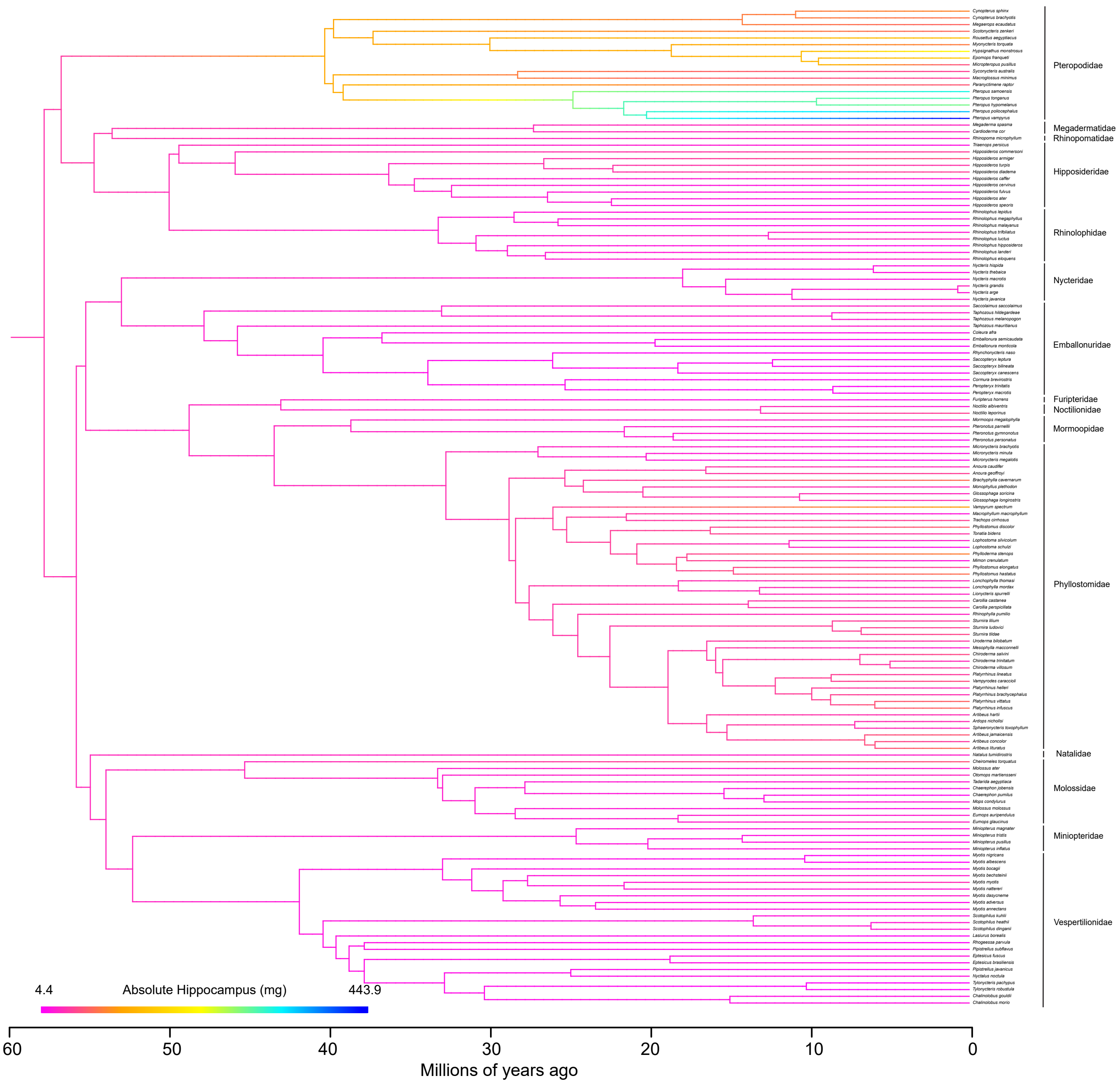

Supplementary Figure 1e.

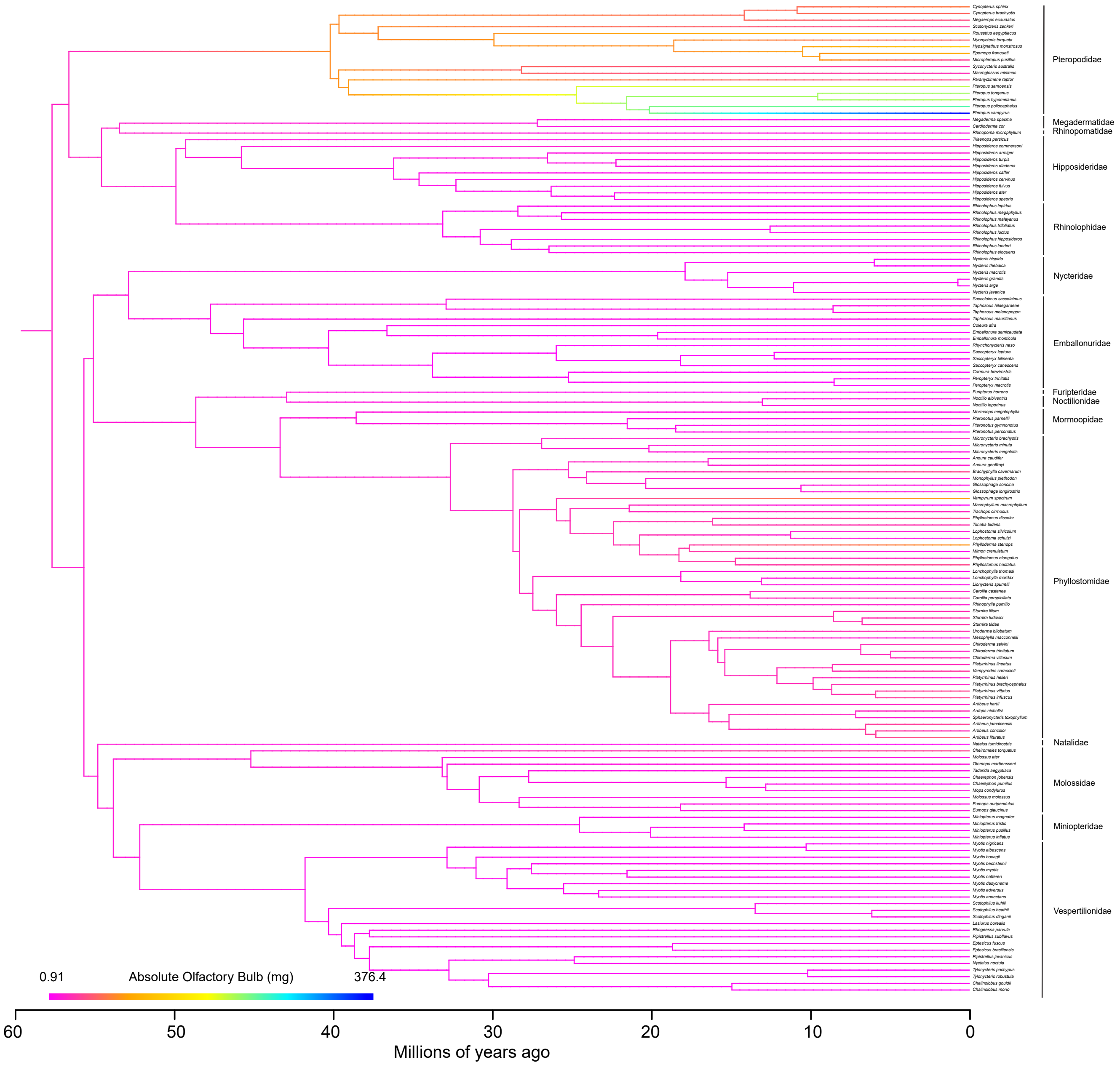

Supplementary Figure 1f.

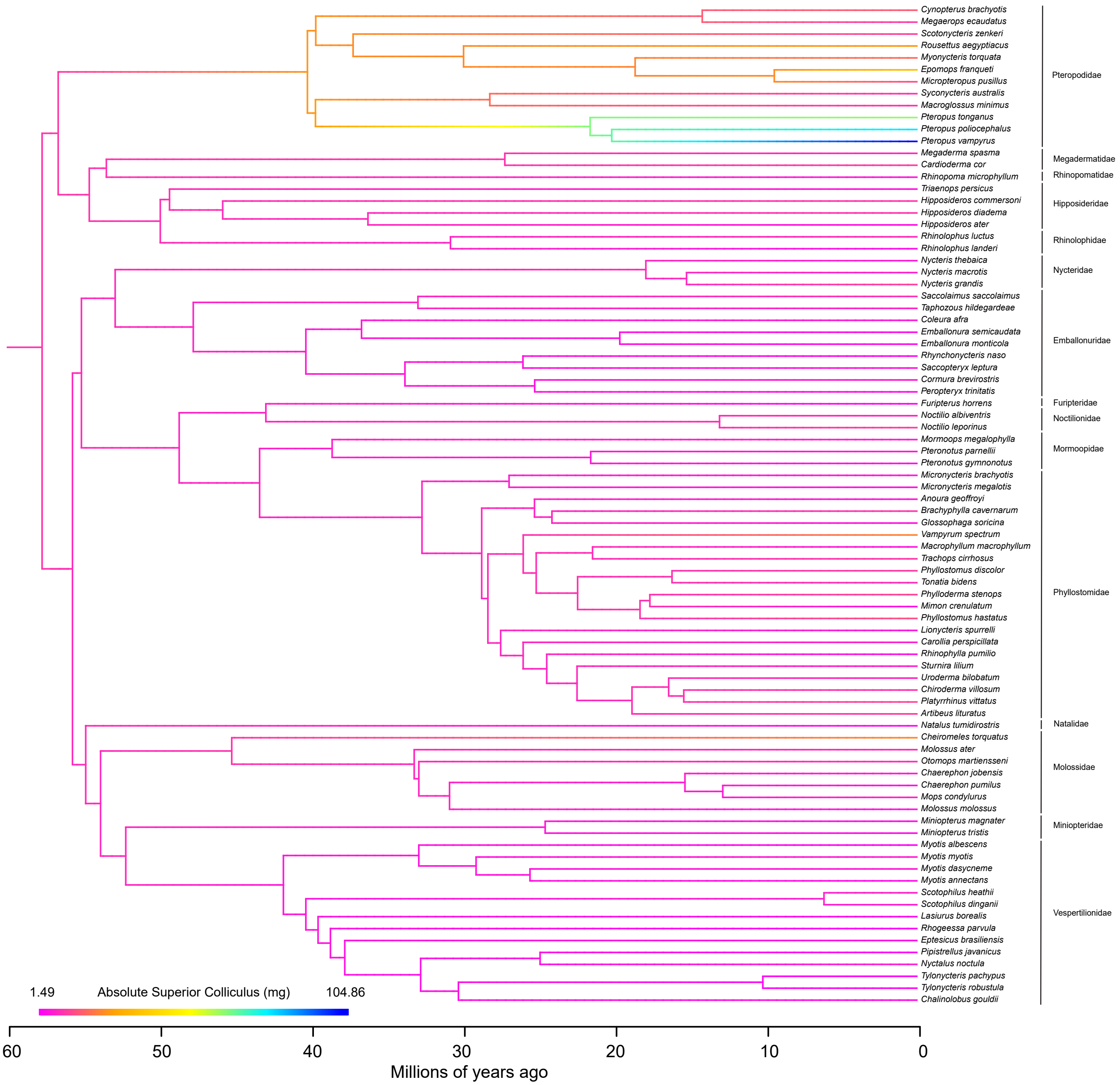

Supplementary Figure 1g.

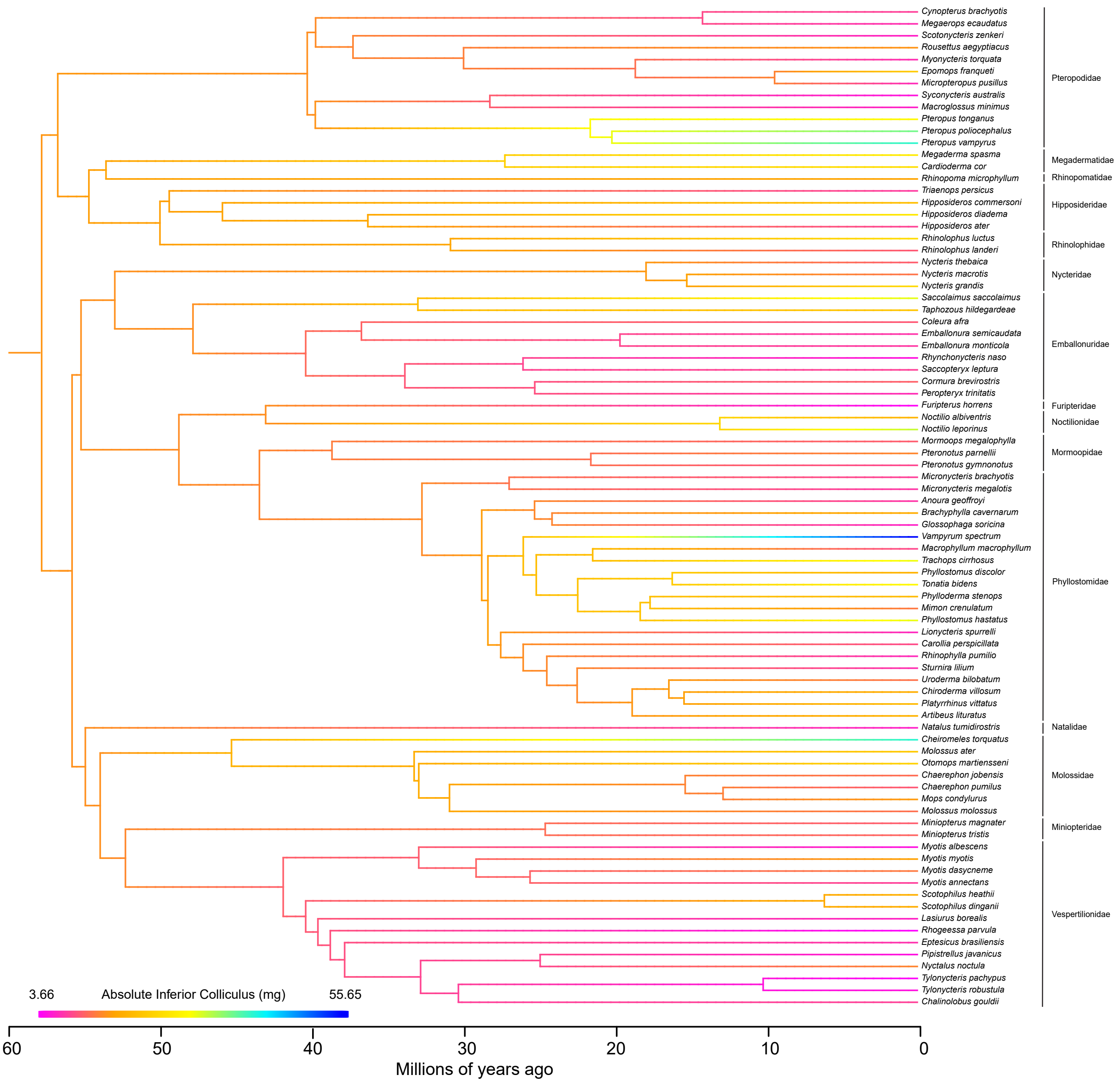

Supplementary Figure 1h.

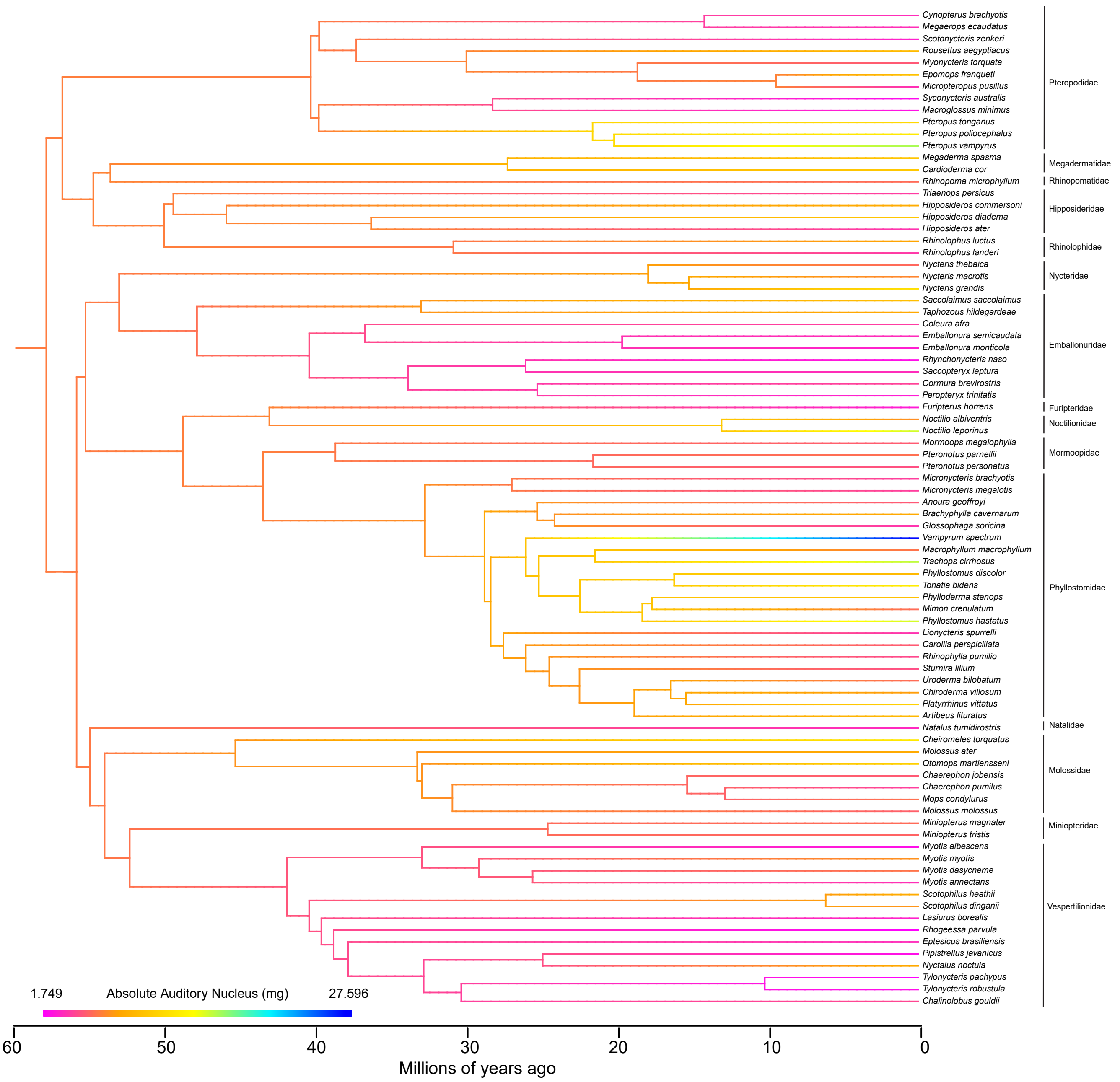

Supplementary Figure 1i.

Supplementary Figure 2. **Phylogenetic reconstructions of mass-corrected brain regions.** Estimates of the maximum likelihood (ML) ancestral states for mass-corrected, (a) eye, (b) brain, and (c-h) brain regions. Phylogenetically informed mass-residuals were generated and mapped onto the tree by estimating states at internal nodes using ML and interpolating the states along each edge. The phylogeny and variation in the residuals were combined to visualize the increase and decrease of these traits in different lineages over evolutionary time. The color gradient indicates trait size, as mapped onto the phylogeny (families indicated along right side).

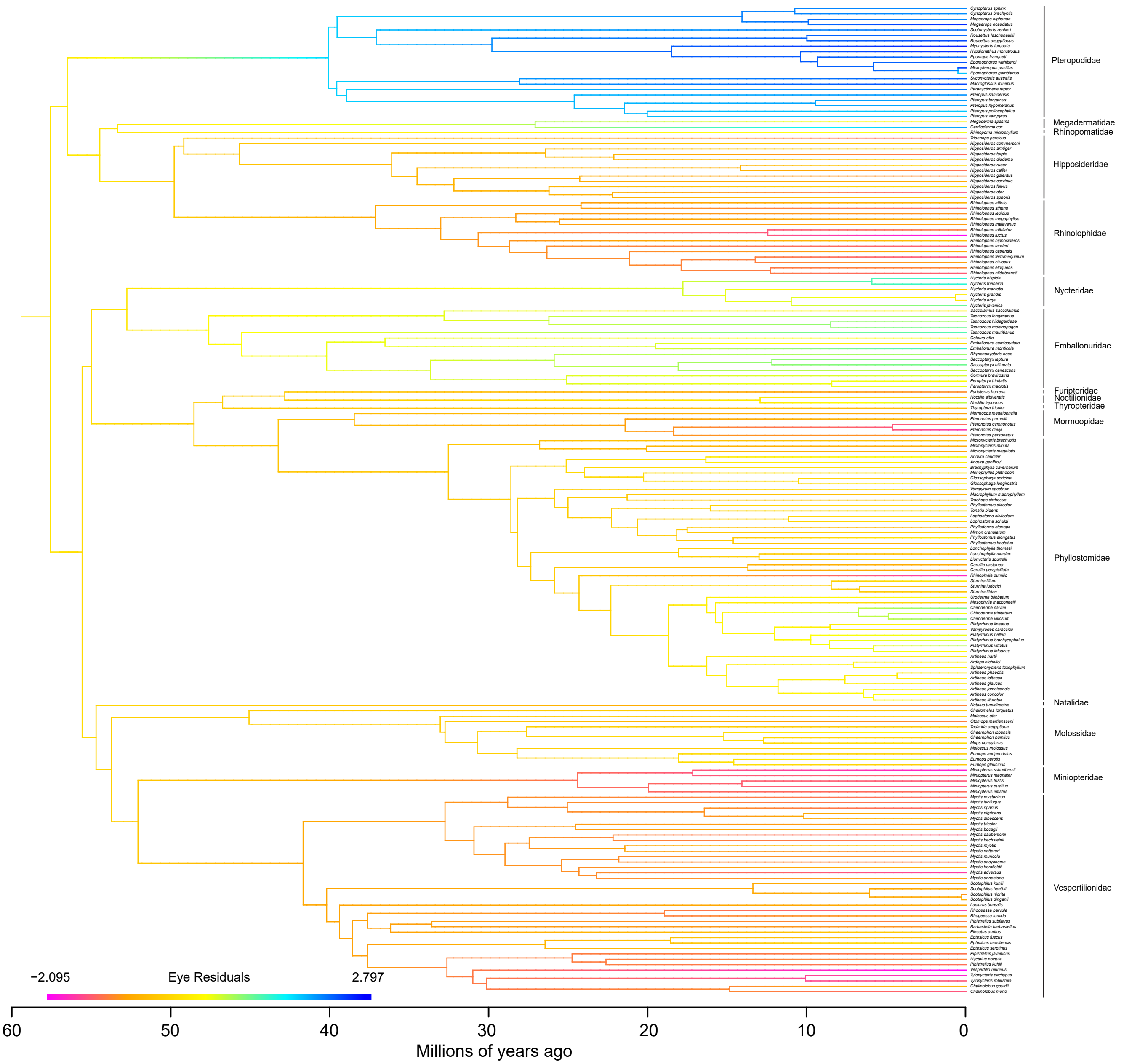

Supplementary Figure 2a.

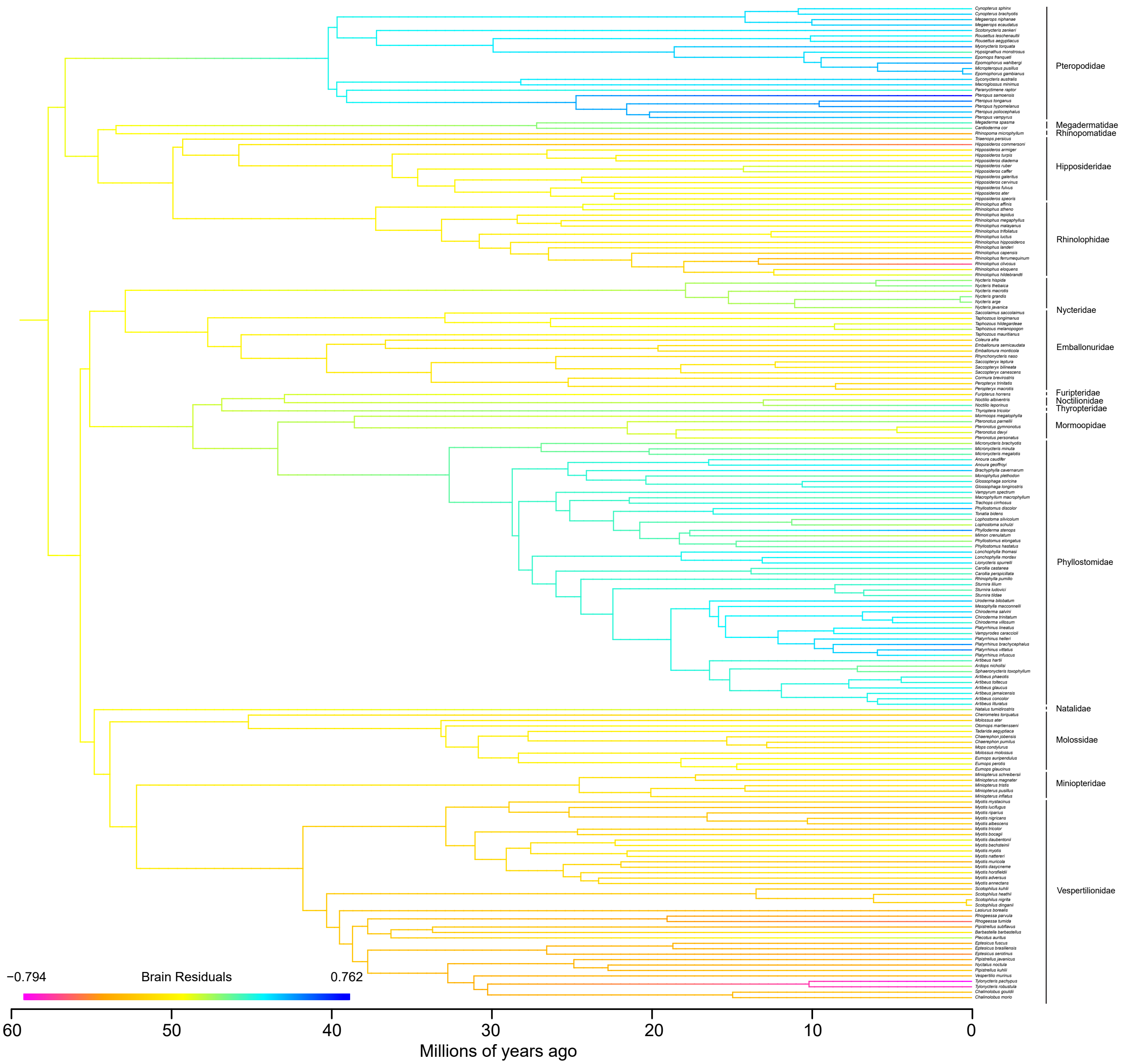

Supplementary Figure 2b.

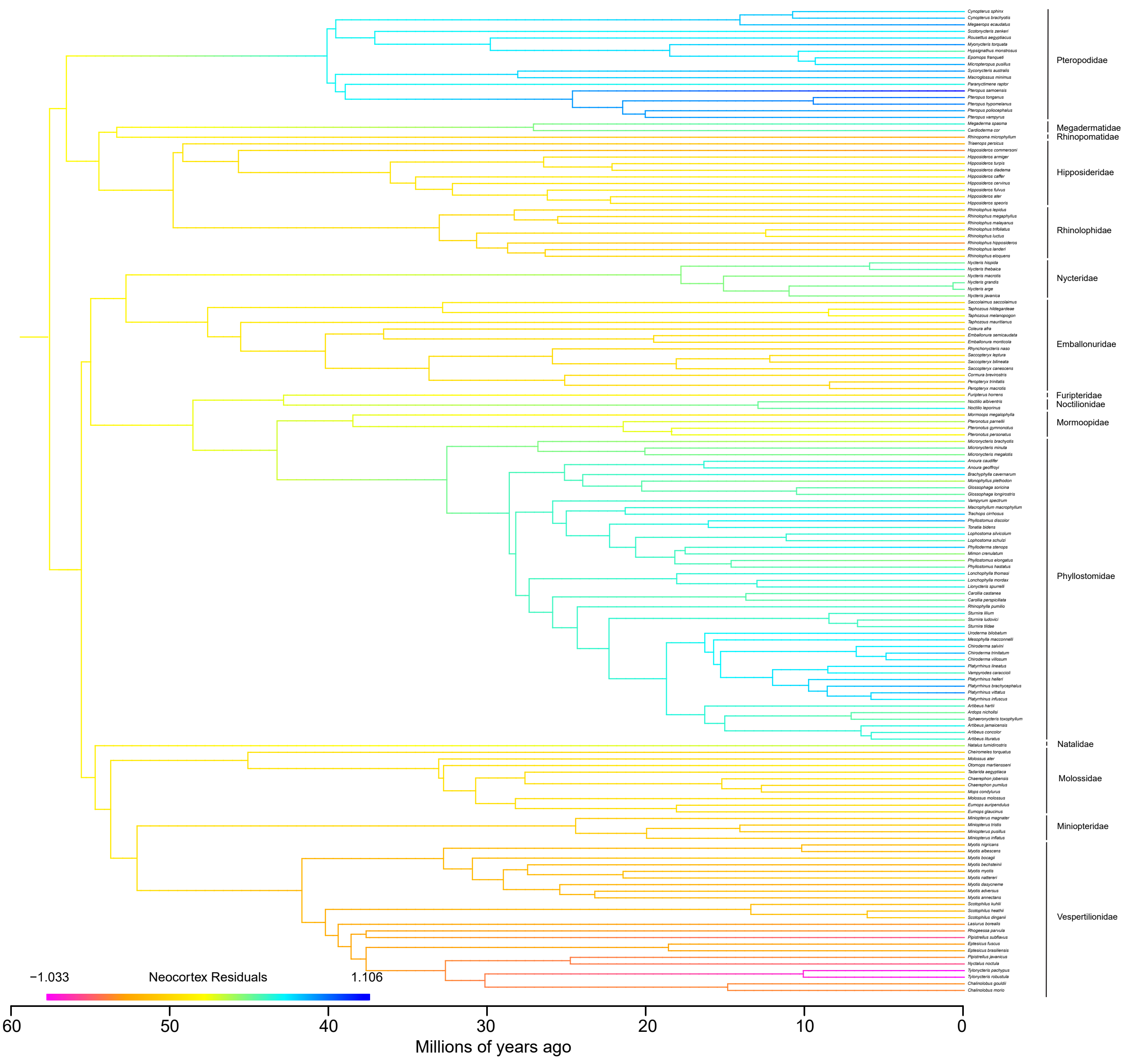

Supplementary Figure 2c.

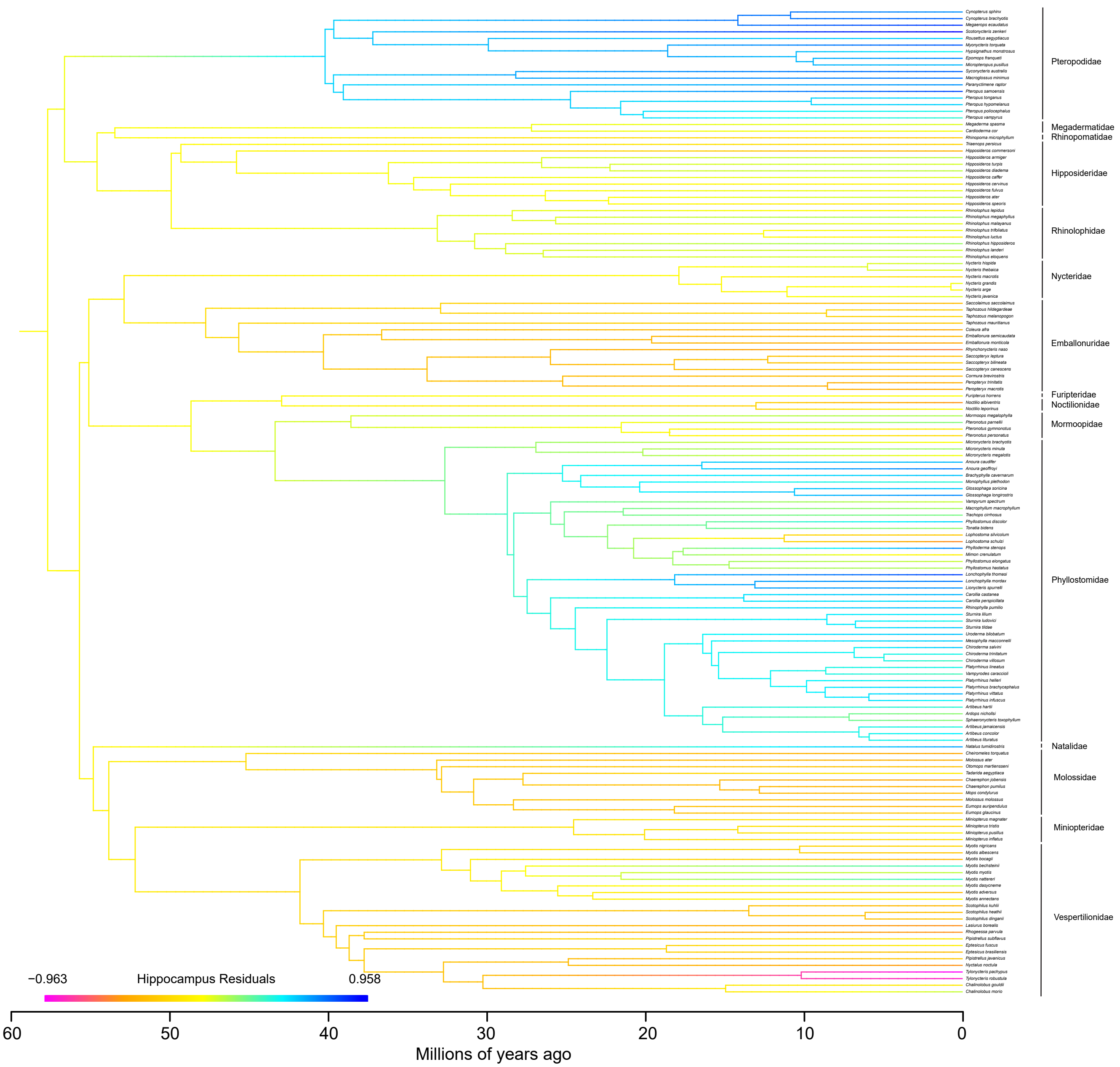

Supplementary Figure 2d.

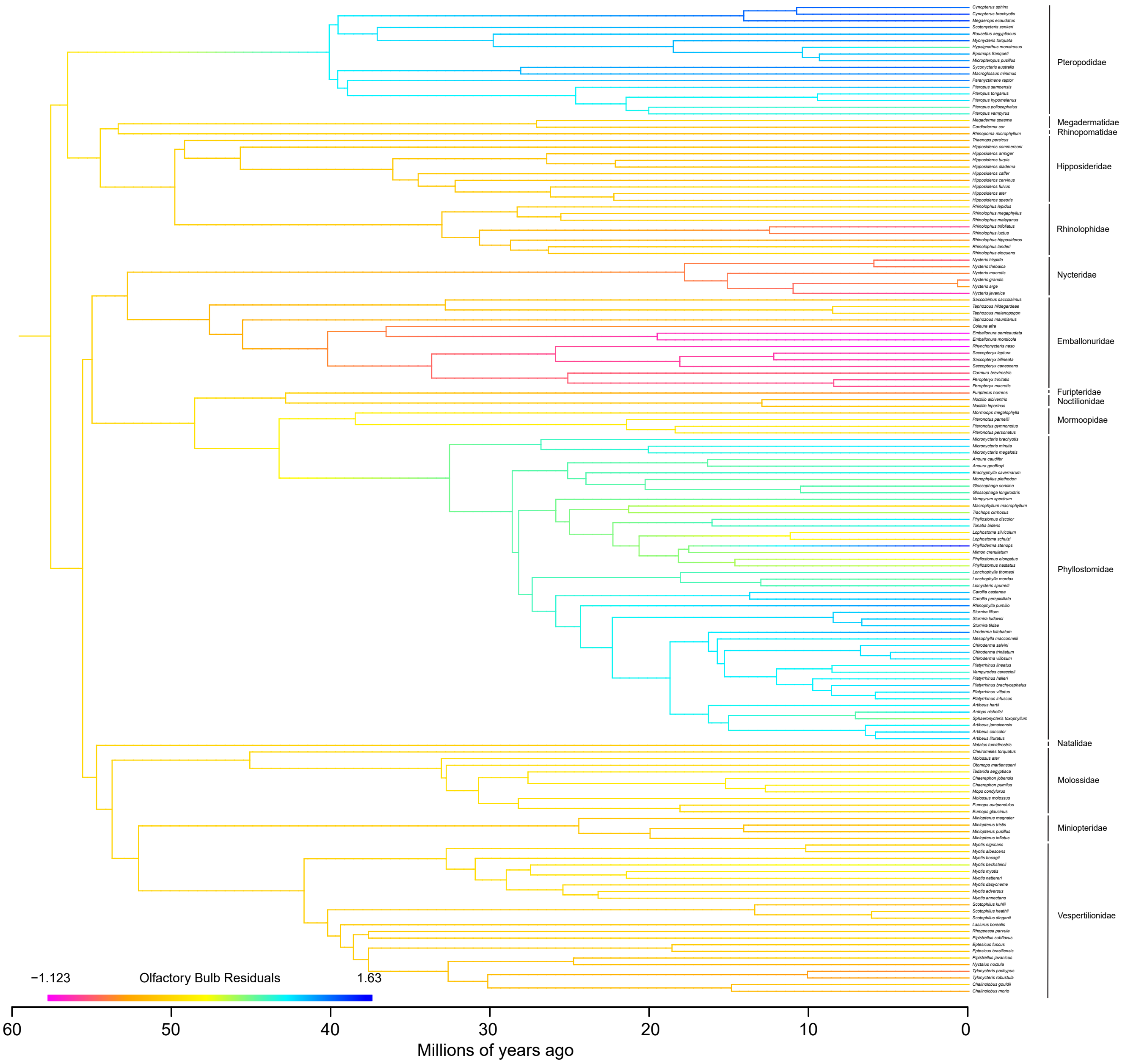

Supplementary Figure 2e.

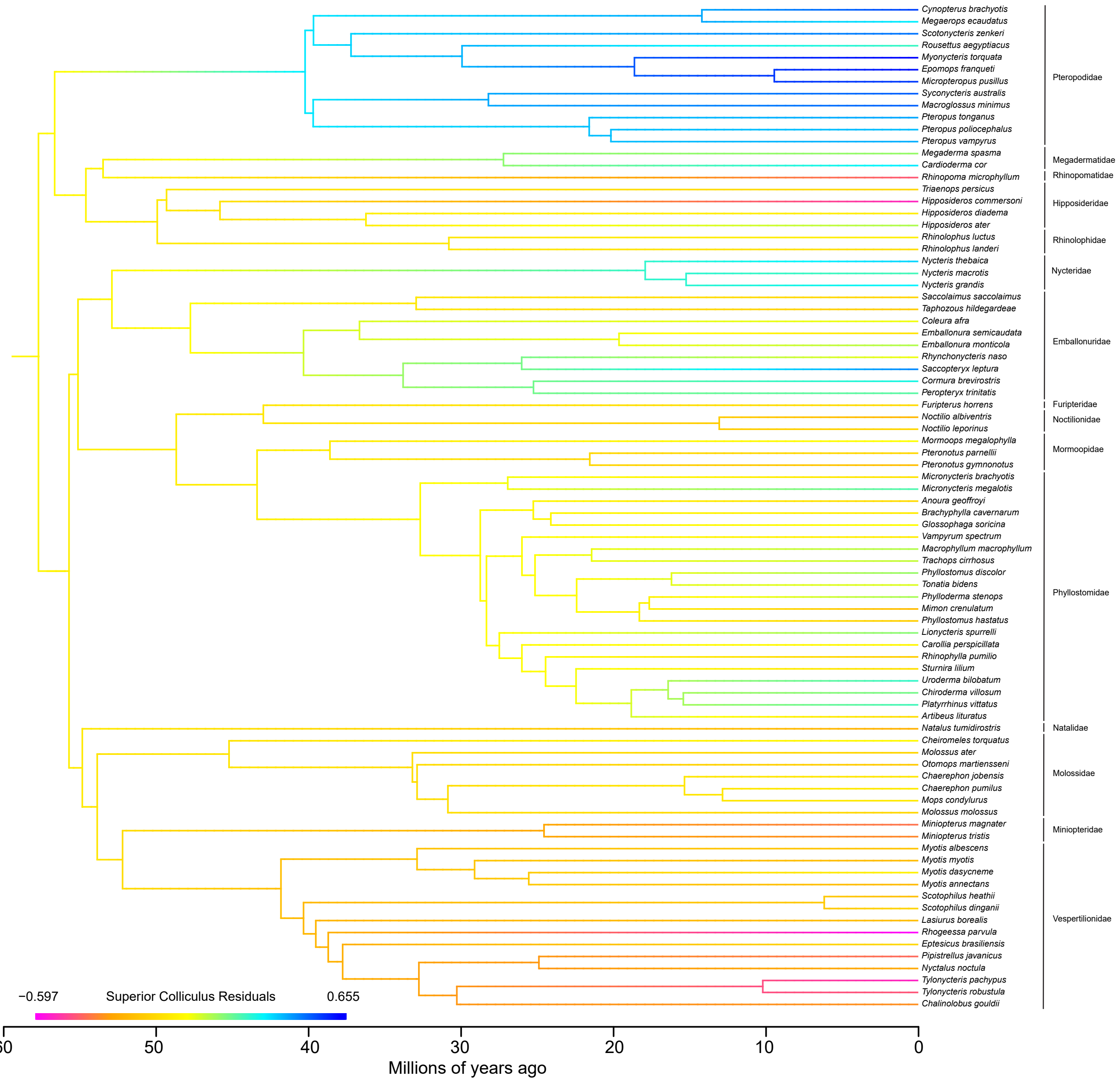

Supplementary Figure 2f.

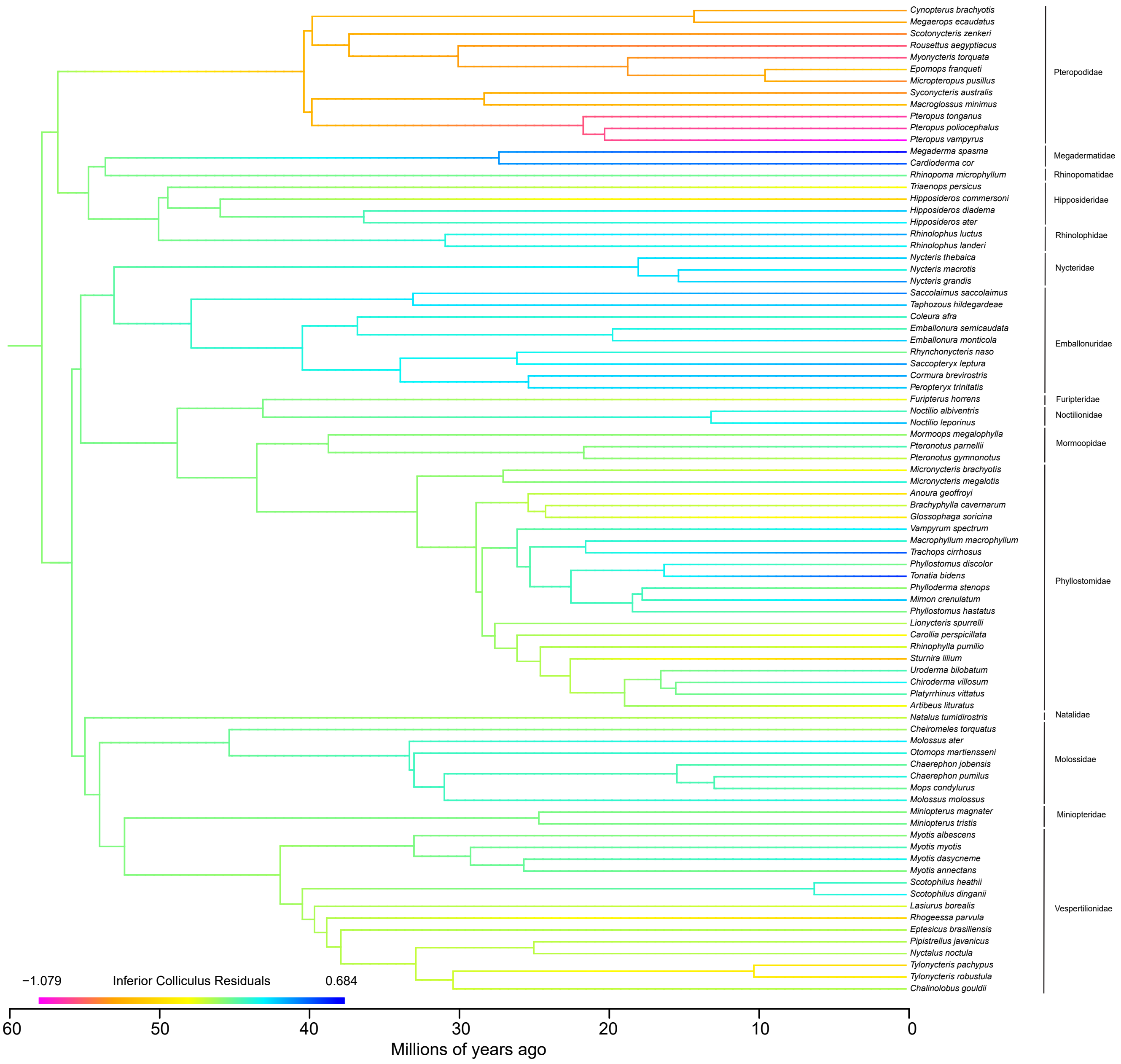

Supplementary Figure 2g.

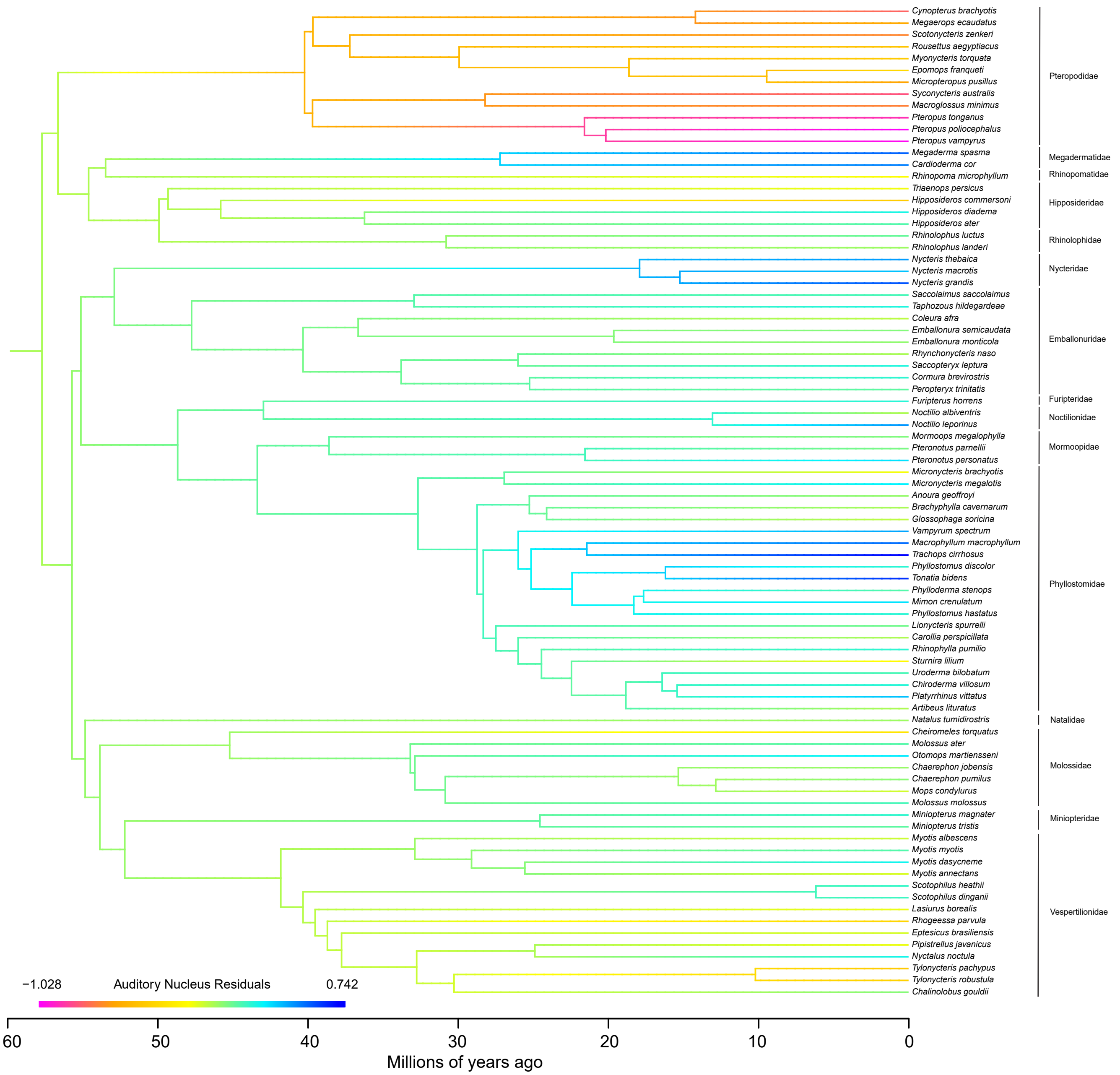

Supplementary Figure 2h.

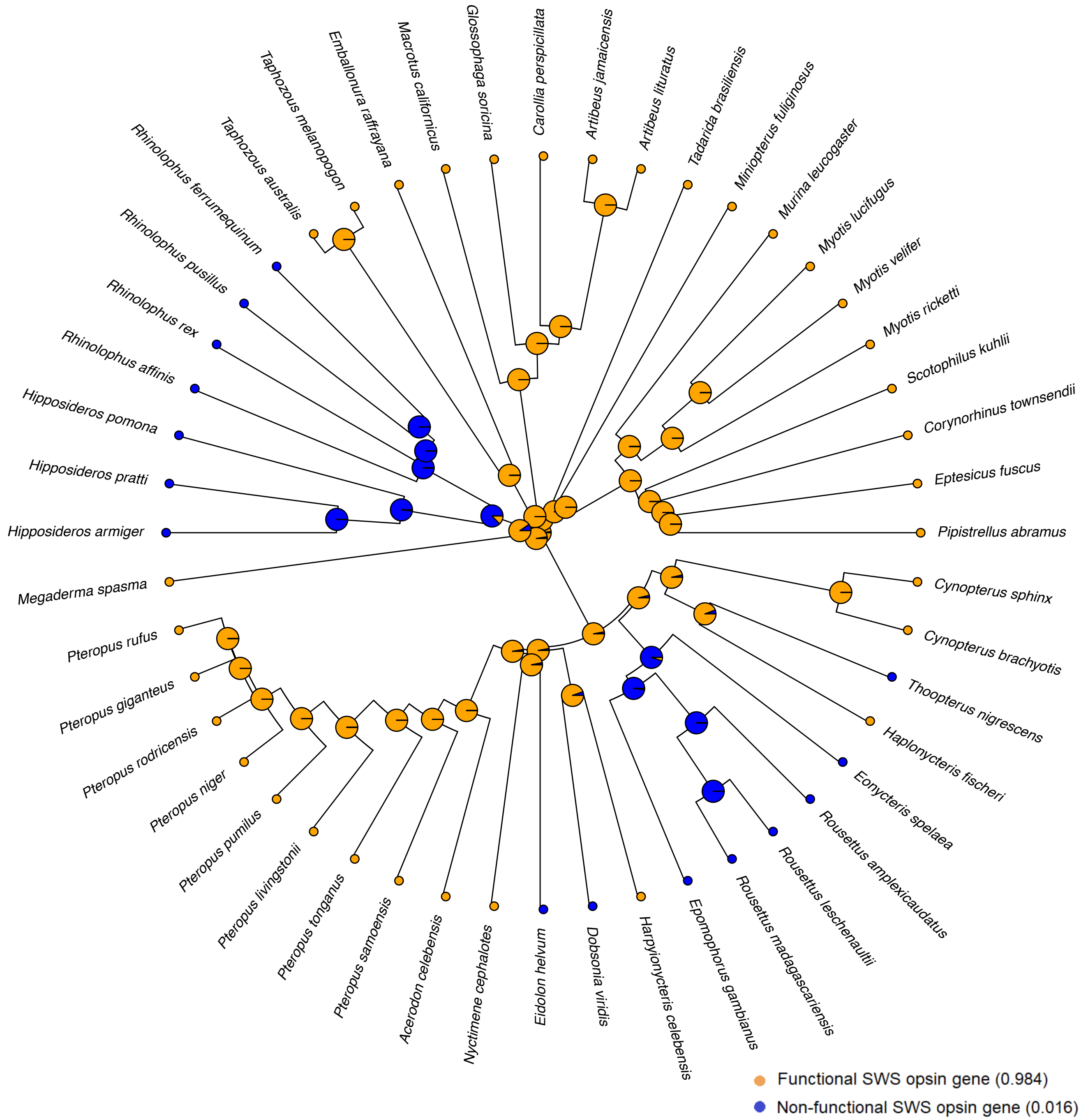

Supplementary Figure 3. The ancestral state estimates of short-wavelength sensitive (SWS) opsin gene functionality. Bat species (*N*= 41) were categorized as having either (i) functional or (ii) non-functional SWS opsin genes and the functionality of the ancestral SWS opsin gene was estimated under an equal rates model of evolution. These marginal ancestral states (i.e. Bayesian posterior probabilities), have been overlain on the phylogeny. We found support for a functional SWS opsin in the ancestral bat (Bayesian posterior probabilities: functional SWS opsin gene: 0.971; non-functional SWS opsin gene: 0.029).

Supplementary Table 1: *Post hoc* comparisons of phylogenetically adjusted data (log transformed body mass, brain volume, eye mass, and brain regions) among foraging strategies (t-values and Holm-Bonferroni corrected p-values in parentheses).

Foraging Strategy	P-LE	P-NLE
A-LE	[a] -1.446 (1.000)	[a] -8.983 (0.03)
	[b] -17.254 (0.003)	[b] -17.254(0.003)
	[c] -4.829 (1.000)	[c] -11.550 (0.003)
	[d] -4.813 (0.302)	[d] -10.121 (0.003)
	[e] -6.544 (1.000)	[e] -12.098 (0.003)
	[f] -7.280 (0.10)	[f] -12.000 (0.003)
	[g] -1.050(0.637)	[g] -7.339 (0.018)
	[h] 0.792 (1.000)	[h] 0.262 (1.000)
	[i] -0.201 (1.000)	[i] 0.179 (1.000)
P-LE		[a] -6.666 (0.21)
		[b] -12.029 (0.012)
		[c] -6.517 (0.218)
		[d] -5.590 (0.302)
		[e] -6.147 (0.164)
		[f] -5.560 (0.160)
		[g] -4.729 (0.120)
		[h] -0.423 (1.000)
		[i] 0.293 (1.000)

[a] Log Mass
[b] Log Eye
[c] Log Brain
[d] Log Neocortex
[e] Log Hippocampus
[f] Log Olfactory Bulb
[g] Log Superior Colliculus
[h] Log Inferior Colliculus
[i] Log Auditory Nucleus

A-LE - Predatory Laryngeal Echolocator
P-LE - Phytophagous Laryngeal Echolocator
P-NLE - Phytophagous Non-Laryngeal Echolocator

Supplementary Table 2: *Post hoc* comparisons of phylogenetically adjusted data (mass residuals of brain volume, eye mass, and brain regions) among foraging strategies (t-values and Holm-Bonferroni corrected p-values in parentheses).

Foraging Strategy	P-LE	P-NLE
A-LE	[a] -4.146 (0.340)	[a] -16.081 (0.003)
	[b] -11.489 (0.012)	[b] -11.633 (0.006)
	[c] -9.641 (0.022)	[c] -9.915 (0.006)
	[d] -12.588 (0.003)	[d] -11.916(0.003)
	[d] -12.703 (0.003)	[d] -12.087(0.003)
	[f] -1.634 (0.456)	[f] -8.438 (0.009)
	[g] 3.370(0.113)	[g] 12.016 (0.003)
	[h] 0.689 (0.751)	[h] 10.775 (0.006)
P-LE		[a] -10.856(0.022)
		[b]-1.982 (0.644)
		[c] -2.143 (0.588)
		[d] -1.897(0.623)
		[e] -1.968 (0.609)
		[f] -5.102 (0.114)
		[g] 6.445(0.028)
		[h] 7.611 (0.008)

[a] Log Eye
[b] Log Brain
[c] Log Neocortex
[d] Log Hippocampus
[e] Log Olfactory Bulb
[f] Log Superior Colliculus
[g] Log Inferior Colliculus
[h] Log Auditory Nucleus

A-LE - Predatory Laryngeal Echolocator
P-LE - Phytophagous Laryngeal Echolocator
P-NLE - Phytophagous Non-Laryngeal Echolocator

Supplementary Table 3: *Post hoc* comparisons of phylogenetically adjusted data (log transformed body mass, brain volume, eye mass, and brain regions) among echolocating call type (t-values and Holm-Bonferroni corrected p-values in parentheses).

CALL TYPE	DH	MH	NLE
**CF**	[a] 1.289 (1.000)	[a] -0.189 (1.000)	[a] -6.911 (0.175)
	[b] 1.127 (0.757)	[b] -4.794 (0.412)	[b] -16.542(0.006)
	[c] 2.112 (1.000)	[c] -2.119 (1.000)	[c] -9.100 (0.025)
	[d] 2.119 (0.906)	[d] -2.236 (0.906)	[d] -8.020 (0.045)
	[e] 2.302 (0.846)	[e] -1.218 (0.846)	[e] -8.167 (0.040)
	[f] 0.272 (1.000)	[f] -2.304 (1.000)	[f] -8.508 (0.030)
	[g] 1.959(0.903)	[g] 0.249 (0.903)	[g] -4.617 (0.085)
	[h] 1.992 (1.000)	[h] 1.002 (1.000)	[h] 1.144 (1.000)
	[i] 1.614 (1.000)	[i] 0.332 (1.000)	[i] 0.782 (1.000)
**DH**		[a] -1.914 (1.000)	[a] -8.873 (0.120)
		[b] -7.390 (0.267)	[b] -19.486 (0.006)
		[c] -5.371 (0.621)	[c] -12.055 (0.024)
		[d] -5.647 (0.420)	[d] -10.942 (0.006)
		[e] -4.637 (0.654)	[e] -11.276 (0.012)
		[f] -3.222 (1.000)	[f] -9.766 (0.030)
		[g] -2.597 (0.903)	[g] -7.925 (0.018)
		[h] -1.573 (1.000)	[h] -0.776 (1.000)
		[i] -1.960 (1.000)	[i] -0.809 (1.000)
**MH**			[a] -7.888 (0.175)
			[b] -15.093 (0.006)
			[c] -8.732 (0.088)
			[d] -7.661 (0.164)
			[e] -8.774 (0.084)
			[f] -8.188 (0.064)
			[g] -6.446 (0.085)
			[h] 0.405 (1.000)
			[i] 0.673(1.000)

[a] Log Mass
[b] Log Eye
[c] Log Brain
[d] Log Neocortex
[e] Log Hippocampus
[f] Log Olfactory Bulb
[g] Log Superior Colliculus
[h] Log Inferior Colliculus
[i] Log Auditory Nucleus

CF - Constant Frequency
DH - Fundamental harmonic frequency modulated
MH - Multi-harmonic
NLE - Non-laryngeal Echolocators

Supplementary Table 4: *Post hoc* comparisons of phylogenetically adjusted data (mass residuals of total brain volume, eye mass, and brain regions) among echolocating call type (t-values and Holm-Bonferroni corrected p-values in parentheses).

CALL TYPE	DH	MH	NLE
CF	[a] 0.334 (0.929)	[a] -6.938 (0.128)	[a] -17.418 (0.006)
	[b] 3.167(0.362)	[b] -6.433 (0.201)	[b] -19.640 (0.028)
	[c] 3.798 (0.246)	[c] -6.365 (0.126)	[c] -19.343 (0.008)
	[d] 2.914 (0.596)	[d] -2.362 (0.596)	[d] -16.403 (0.090)
	[e] 0.659 (0.815)	[e] -3.826 (0.693)	[e] -7.532 (0.030)
	[f] 1.301 (0.477)	[f] -2.772 (0.214)	[f] -7.786 (0.006)
	[g] 1.325 (1.000)	[g] -0.008 (1.000)	[g] 8.245 (0.006)
	[h] 0.462 (0.800)	[h] -1.769 (0.657)	[h] 7.416 (0.006)
DH		[a] -9.005 (0.099)	[a] -19.754(0.006)
		[b] -12.074 (0.010)	[b] -13.602 (0.006)
		[c] 13.042 (0.006)	[c] -13.985(0.006)
		[d] -6.884(0.286)	[d] -9.874 (0.012)
		[e] -3.840 (0.693)	[e] -7.808 (0.693)
		[f] -5.911 (0.069)	[f] -11.150 (0.006)
		[g] -2.007 (1.000)	[g] 8.819 (0.012)
		[h] -3.217 (0.657)	[h] 8.739 (0.012)
MH			[a] -14.163 (0.008)
			[b] -5.545 (0.362)
			[c] -5.465 (0.246)
			[d] -5.585 (0.351)
			[e] -5.607(0.380)
			[f] -7.295 (0.040)
			[g] 11.022 (0.006)
			[h] 11.888 (0.006)

[a] Log Eye
[b] Log Brain
[c] Residuals (Log Neocortex Log Mass)
[d] Residuals (Log Hippocampus Log Mass)
[e] Residuals (Log Olfactory Bulb Log Mass)
[f] Residuals (Log Superior Colliculus Log Mass)
[g] Residuals (Log Inferior Colliculus Log Mass)
[h] Residuals (Log Auditory Nucleus Log Mass)

CF - Constant Frequency
DH - Fundamental harmonic frequency modulated calls
MH - Multi-Harmonic
NLE - Non-laryngeal Echolocators

Supplementary Table 5: *Post hoc* comparisons of phylogenetically adjusted data (log transformed body mass, brain volume, eye mass, and brain regions) among call type in predatory bats (t-values and Holm-Bonferroni corrected p-values in parentheses).

CALL TYPE	DH	MH
**CF**	[a] 1.423 (1.000)	[a] -0.209 (1.000)
	[b] 1.118 (0.739)	[b] -4.754 (0.386)
	[c] 2.273 (1.000)	[c] -2.281 (1.000)
	[d] 2.262 (0.802)	[d] -2.387 (0.802)
	[e] 2.376 (0.792)	[e] -1.258 (0.792)
	[f] 0.269 (1.000)	[f] -2.281(1.000)
	[g] 2.167(0.672)	[g] 0.275 (0.867)
	[h] 2.083 (0.735)	[h] 1.048(1.000)
	[i] 1.692 (1.000)	[i] 0.348 (1.000)
**DH**		[a] -2.113 (1.000)
		[b] -7.329 (0.300)
		[c]-5.780 (0.495)
		[d] -6.029 (0.333)
		[e] -4.786(0.591)
		[f] -3.189 (1.000)
		[g] -2.871 (0.672)
		[h] -1.644 (1.000)
		[i] -2.054 (1.000)

[a] Log Mass
[b] Log Eye
[c] Log Brain
[d] Log Neocortex
[e] Log Hippocampus
[f] Log Olfactory Bulb
[g] Log Superior Colliculus
[h] Log Inferior Colliculus
[i] Log Auditory Nucleus

CF - Constant Frequency
DH - Fundamental harmonic frequency modulated
MH - Multi-harmonic

Supplementary Table 6: *Post hoc* comparisons of phylogenetically adjusted data (mass residuals of total brain volume, eye mass, and brain regions) among call type in predatory bats (t-values and Holm-Bonferroni corrected p-values in parentheses).

CALL TYPE	DH	MH
CF	[a] 0.229 (0.940)<br>[b] 3.048 (0.370)<br>[c] 3.660 (0.192)<br>[d] 2.769 (0.708)<br>[e] -0.688 (0.829)<br>[f] 1.352 (0.495)<br>[g] 1.006 (1.000)<br>[h] 0.240 (0.883)	[a] -6.669 (0.129)<br>[b] -6.144 (0.182)<br>[c] -6.175 (0.090)<br>[d] -2.262 (0.708)<br>[e] -3.697 (0.732)<br>[f] -2.585 (0.280)<br>[g] -0.206 (1.000)<br>[h] -1.972 (0.693)
DH	`	[a] -8.536 (0.129)<br>[b] -11.562 (0.182)<br>[c] -12.619 (0.006)<br>[d] -6.563 (0.252)<br>[e] -3.641 (0.732)<br>[f] -5.722 (0.090)<br>[g] -1.810 (1.000)<br>[h] -3.172 (0.693)

[a] Log Eye
[b] Log Brain
[c] Residuals (Log Neocortex Log Mass)
[d] Residuals (Log Hippocampus Log Mass)
[e] Residuals (Log Olfactory Bulb Log Mass)
[f] Residuals (Log Superior Colliculus Log Mass)
[g] Residuals (Log Inferior Colliculus Log Mass)
[h] Residuals (Log Auditory Nucleus Log Mass)

CF - Constant Frequency
DH - Fundamental harmonic frequency modulated calls
MH - Multi-Harmonic

Supplementary Table 7. Species categorized as having either a functional (Yes) or non-functional (No) short-wave opsin (SWS) gene. Asterisk (*) indciates species in our eyesize dataset. Bold Lettering indicates predatory species used in binary CF versus non-CF test.

**Family**	**Species**	**Functional SWS**
Emballonuridae	***Emballonura raffrayana***	Yes
Emballonuridae	***Taphozous australis***	Yes
Emballonuridae	****Taphozous melanopogon***	Yes
Hipposideridae	****Hipposideros armiger***	No
Hipposideridae	***Hipposideros pomona***	No
Hipposideridae	***Hipposideros pratti***	No
Megadermatidae	****Megaderma spasma***	Yes
Miniopteridae	***Miniopterus fuliginosus***	Yes
Phyllostomidae	**Artibeus jamaicensis*	Yes
Phyllostomidae	**Artibeus lituratus*	Yes
Phyllostomidae	**Carollia perspicillata*	Yes
Phyllostomidae	**Glossophaga soricina*	Yes
Pteropodidae	*Acerodon celebensis*	Yes
Pteropodidae	**Cynopterus brachyotis*	Yes
Pteropodidae	*Dobsonia viridis*	No
Pteropodidae	*Eidolon helvum*	No
Pteropodidae	*Eonycteris spelaea*	No
Pteropodidae	**Epomophorus gambianus*	No
Pteropodidae	*Haplonycteris fischeri*	Yes
Pteropodidae	*Harpyionycteris celebensis*	Yes
Pteropodidae	*Nyctimene cephalotes*	Yes
Pteropodidae	*Pteropus giganteus*	Yes
Pteropodidae	*Pteropus livingstonii*	Yes
Pteropodidae	*Pteropus niger*	Yes
Pteropodidae	*Pteropus pumilus*	Yes
Pteropodidae	*Pteropus rodricensis*	Yes
Pteropodidae	*Pteropus rufus*	Yes
Pteropodidae	**Pteropus samoensis*	Yes
Pteropodidae	**Pteropus tonganus*	Yes
Pteropodidae	*Rousettus amplexicaudatus*	No
Pteropodidae	*Rousettus madagascariensis*	No
Pteropodidae	*Thoopterus nigrescens*	No
Rhinolophidae	****Rhinolophus affinis***	No
Rhinolophidae	****Rhinolophus ferrumequinu***	No
Rhinolophidae	***Rhinolophus pusillus***	No
Rhinolophidae	***Rhinolophus rex***	No
Vespertilionidae	***Murina leucogaster***	Yes
Vespertilionidae	****Myotis lucifugus***	Yes
Vespertilionidae	***Myotis ricketti***	Yes
Vespertilionidae	***Myotis velifer***	Yes
Vespertilionidae	***Pipistrellus abramus***	Yes

Supplementary Table 8. Specimen numbers for the skulls from Royal Ontario Museum (ROM) or Museum of Natural History in Denmark (MNHD).

Species	Specimen #	Source
Anoura caudifer	91179	ROM
	70909	ROM
	78418	ROM
Anoura geoffroyi	78489	ROM
	78494	ROM
	78490	ROM
Ardops nichollsi	71467	ROM
	71463	ROM
Artibeus concolor	47110	ROM
	59924	ROM
Artibeus glaucus	51851	ROM
	53609	ROM
	51853	ROM
Artibeus hartii	84977	ROM
Artibeus jamaicensis	95323	ROM
	95271	ROM
	95325	ROM
Artibeus lituratus	33319	ROM
	33296	ROM
	33297	ROM
Artibeus phaeotis	33595	ROM
	33541	ROM
	33591	ROM
Artibeus toltecus	85961	ROM
	85891	ROM
Barbastella barbastellus	78690	ROM
	78691	ROM
Brachyphylla cavernarum	74396	ROM
	74397	ROM
Cardioderma cor	91505	ROM
	91507	ROM
Carollia castanea	44975	ROM
	90111	ROM
Carollia perspicillata	78672	ROM
	58717	ROM
	58716	ROM
Chalinolobus gouldii	78355	ROM
	102789	ROM
Chalinolobus morio	58403	ROM
	102788	ROM
Chaerephon jobensis	696	MNHD
Chaerephon pumilus	CN2326	MNHD
	CN2324	MNHD
	CN2323	MNHD
	CN2321	MNHD
Cheiromeles torquatus	44150	ROM
	38039	ROM
Chiroderma salvini	78472	ROM
	91194	ROM
Chiroderma trinitatum	78692	ROM

Species	Specimen #	Source
Myotis myotis	35341	ROM
	91218	ROM
	35343	ROM
Myotis mystacinus	94103	ROM
	102816	ROM
Myotis nattereri	KAT6.31132	MNHD
	KAT5.7436	MNHD
Myotis nigricans	78791	ROM
	78781	ROM
Myotis riparius	103988	ROM
	104066	ROM
Myotis tricolor	71049	ROM
	78629	ROM
Natalus tumidirostris	31434	ROM
	31451	ROM
Noctilio albiventris	78011	ROM
Noctilio leporinus	940	MNHD
Nyctalus noctula	SR16	MNHD
	L39.3075	MNHD
	KAT22.21634	MNHD
	CHVVE12	MNHD
Nycteris arge	80317	ROM
	46512	ROM
Nycteris grandis	56109	ROM
	56256	ROM
Nycteris hispida	3191	MNHD
	3155	MNHD
	14702	MNHD
	1471	MNHD
	1054	MNHD
Nycteris macrotis	91174	ROM
	73249	ROM
Nycteris javanica	L858.3089	MNHD
	99	MNHD
Nycteris thebaica	40275	ROM
Otomops martiensseni	36520	ROM
	36523	ROM
Paranyctimene raptor	72906	ROM
	58429	ROM
Peropteryx macrotis	L54	MNHD
	546	MNHD
Peropteryx trinitatis	107831	ROM
	107922	ROM
Phylloderma stenops	47455	ROM
	35244	ROM
Phyllostomus discolor	75053	ROM
	44869	ROM
Phyllostomus elongatus	105673	ROM
	57249	ROM
Phyllostomus hastatus	32420	ROM

	96246	ROM		105306	ROM
	97461	ROM		105358	ROM
Chiroderma villosum	45243	ROM	*Pipistrellus javanicus*	86451	ROM
	45251	ROM	*Pipistrellus kuhlii*	46596	ROM
Coleura afra	36251	ROM		40145	ROM
	36250	ROM	*Pipistrellus subflavus*	76304	ROM
Cormura brevirostris	105746	ROM		76299	ROM
	97983	ROM	*Platyrrhinus brachycephalus*	67483	ROM
Cynopterus brachyotis	48089	ROM		67501	ROM
	46347	ROM	*Platyrrhinus helleri*	94252	ROM
	48088	ROM		94222	ROM
Cynopterus sphinx	LXX.0099	MNHD	*Platyrrhinus infuscus*	63225	ROM
Emballonura monticola	38770	ROM		75079	ROM
	38769	ROM	*Platyrrhinus lineatus*	78457	ROM
Emballonura semicaudata	82967	ROM		70919	ROM
	79915	ROM	*Platyrrhinus vittatus*	97305	ROM
Epomophorus gambianus	56069	ROM		97306	ROM
Epomophorus wahlbergi	41785	ROM	*Plecotus auritus*	KAT8.11533	MNHD
	41786	ROM		KAT7.12431PA	MNHD
	41789	ROM		CHVE6	MNHD
Epomops franqueti	43331	ROM	*Pteronotus davyi*	96495	ROM
	43334	ROM		96500	ROM
Eptesicus brasiliensis	98114	ROM	*Pteronotus gymnonotus*	107925	ROM
Eptesicus fuscus	78288	ROM		113957	ROM
	78176	ROM	*Pteronotus parnellii*	101424	ROM
	78281	ROM		101476	ROM
Eptesicus serotinus	78317	ROM		101360	ROM
	78313	ROM	*Pteronotus personatus*	45111	ROM
Eumops auripendulus	53965	ROM		45110	ROM
	50195	ROM	*Pteropus hypomelanus*	51337	ROM
Eumops glaucinus	57828	ROM		48126	ROM
	57826	ROM		51343	ROM
Eumops perotis	50201	ROM	*Pteropus poliocephalus*	58396	ROM
	50953	ROM	*Pteropus samoensis*	CN2401	MNHD
Furipterus horrens	104058	ROM		CN2400	MNHD
	100202	ROM	*Pteropus tonganus*	CN2381	MNHD
Glossophaga longirostris	34099	ROM	*Pteropus vampyrus*	48068	ROM
	33841	ROM		48067	ROM
	34063	ROM	*Rhinolophus affinis*	36736	ROM
Glossophaga soricina	90154	ROM		31193	ROM
	90155	ROM	*Rhinolophus capensis*	LXX.0098	MNHD
Hipposideros armiger	38746	ROM	*Rhinolophus clivosus*	L59.3167	MNHD
	38741	ROM		L57.3116	MNHD
Hipposideros ater	40734	ROM	*Rhinolophus eloquens*	81348	ROM
Hipposideros caffer	50911	ROM		36314	ROM
	46812	ROM	*Rhinolophus ferrumequinum*	77744	ROM
Hipposideros cervinus	44063	ROM		77747	ROM
	40968	ROM	*Rhinolophus hildebrandti*	64673	ROM
Hipposideros commersoni	68988	ROM		64481	ROM
	56160	ROM	*Rhinolophus hipposideros*	35335	ROM
Hipposideros diadema	41409	ROM		35334	ROM
	41408	ROM	*Rhinolophus landeri*	50923	ROM
Hipposideros fulvus	104571	ROM		56104	ROM
	104573	ROM	*Rhinolophus lepidus*	L58.3276	MNHD

Hipposideros galeritus	43716	ROM	*Rhinolophus luctus*	40908	ROM
	38197	ROM		86309	ROM
Hipposideros ruber	100523	ROM	*Rhinolophus malayanus*	31188	ROM
	100518	ROM		31184	ROM
Hipposideros speoris	104575	ROM	*Rhinolophus megaphyllus*	77791	ROM
	104574	ROM	*Rhinolophus stheno*	36760	ROM
Hipposideros turpis	77920	ROM		36764	ROM
Hypsignathus monstrosus	56169	ROM	*Rhinolophus trifoliatus*	40977	ROM
	55629	ROM		83983	ROM
Lasiurus borealis	17911	ROM	*Rhinophylla pumilio*	105883	ROM
	20996	ROM		105879	ROM
Lionycteris spurrelli	44228	ROM	*Rhinopoma microphyllum*	L57.3113	MNHD
	45229	ROM		2847	MNHD
Lonchophylla mordax	105798	ROM		2845	MNHD
	91189	ROM	*Rhogeessa parvula*	91235	ROM
Lonchophylla thomasi	105610	ROM	*Rhogeessa tumida*	94223	ROM
	105750	ROM		101321	ROM
Lophostoma schulzi	67468	ROM	*Rhynchonycteris naso*	33000	ROM
	114115	ROM		98769	ROM
Lophostoma silvicolum	100949	ROM		98016	ROM
	105913	ROM	*Rousettus aegyptiacus*	2284	MNHD
Macroglossus minimus	L858.3087	MNHD	*Rousettus leschenaultii*	77958	ROM
Macrophyllum macrophyllum	98614	ROM		77957	ROM
	104389	ROM	*Saccolaimus saccolaimus*	39327	ROM
Megaderma spasma	L858.3085	MNHD		39330	ROM
Megaerops ecaudatus	38734	ROM	*Saccopteryx bilineata*	94243	ROM
	38732	ROM		105362	ROM
Megaerops niphanae	78115	ROM		105672	ROM
Mesophylla macconnelli	63259	ROM	*Saccopteryx canescens*	107890	ROM
Micronycteris brachyotis	98224	ROM		31849	ROM
	98223	ROM	*Saccopteryx leptura*	31609	ROM
Micronycteris megalotis	104440	ROM		59654	ROM
	106132	ROM	*Scotonycteris zenkeri*	100505	ROM
	105788	ROM		54905	ROM
Micronycteris minuta	91172	ROM	*Scotophilus dinganii*	71991	ROM
	84984	ROM		71831	ROM
Micropteropus pusillus	55908	ROM	*Scotophilus heathii*	37373	ROM
	56021	ROM		37349	ROM
Mimon crenulatum	105754	ROM	*Scotophilus kuhlii*	39811	ROM
	105539	ROM		39810	ROM
Miniopterus inflatus	69062	ROM	*Scotophilus nigrita*	76554	ROM
	68941	ROM		76539	ROM
Miniopterus magnater	87615	ROM	*Sphaeronycteris toxophyllum*	88928	ROM
	72902	ROM	*Sturnira lilium*	33540	ROM
Miniopterus pusillus	88412	ROM	*Sturnira ludovici*	106231	ROM
	88411	ROM		99687	ROM
Miniopterus schreibersii	35675	ROM	*Sturnira tildae*	97994	ROM
	68530	ROM		100981	ROM
Miniopterus tristis	67603	ROM	*Syconycteris australis*	87610	ROM
	39861	ROM		87613	ROM
Molossus ater	674	MNHD		87611	ROM
	587	MNHD	*Tadarida aegyptiaca*	78126	ROM
	581	MNHD		48560	ROM
Molossus molossus	920	MNHD	*Taphozous hildegardeae*	74469	ROM

	687	MNHD		74473	ROM
	598	MNHD	*Taphozous longimanus*	37971	ROM
Monophyllus plethodon	74345	ROM		37969	ROM
	74346	ROM	*Taphozous mauritianus*	36265	ROM
Mops condylurus	1507	MNHD		36262	ROM
Mormoops megalophylla	98445	ROM	*Taphozous melanopogon*	550	MNHD
	44925	ROM	*Thyroptera tricolor*	113925	ROM
Myonycteris torquata	36562	ROM		113956	ROM
	36566	ROM	*Tonatia bidens*	56625	ROM
Myotis adversus	58424	ROM		100258	ROM
Myotis albescens	33006	ROM	*Trachops cirrhosus*	105639	ROM
	31885	ROM		105273	ROM
Myotis annectans	86359	ROM	*Triaenops persicus*	73160	ROM
	86358	ROM		68191	ROM
Myotis bechsteinii	L935.3595	MNHD	*Tylonycteris pachypus*	38169	ROM
	CHVE5	MNHD		38170	ROM
Myotis bocagii	57158	ROM	*Tylonycteris robustula*	38164	ROM
	100492	ROM		38166	ROM
Myotis dasycneme	79256	ROM	*Uroderma bilobatum*	105204	ROM
Myotis daubentonii	39635	ROM		105930	ROM
Myotis horsfieldii	36202	ROM	*Vampyrodes caraccioli*	113918	ROM
	38004	ROM	*Vampyrum spectrum*	45335	ROM
Myotis lucifugus	38239	ROM		105870	ROM
	14852	ROM		55915	ROM
	88917	ROM		55902	ROM
Myotis muricola	38002	ROM	*Vespertilio murinus*	LXX.5854	MNHD
	40009	ROM		LXX.5853	MNHD
				L937.3210	MNHD
				CHVE11	MNHD
