# Supplementary material for: Auditory opportunity and visual constraint enabled the evolution of echolocation in bats
Source: Nat Commun. 2018 Jan 8;9:98. doi: 10.1038/s41467-017-02532-x (PMC5758785; doi:10.1038/s41467-017-02532-x)
Supplement: Supplementary file 2 — Description of Additional Supplementary Files [file 41467_2017_2532_MOESM2_ESM.pdf]

File Name: Supplementary Data 1
Description: Species, trait values, and categorizations by call type (MH: multiharmonic, CF: constant frequency, DH: Fundamental harmonic frequency modulated, NLE: non-laryngeal echolocator), diet(A: animal eating, P: phytophagous), echolocation ability (LE: laryngeal echolocator, NLE: nonlaryngeal echolocator) and roost type (I: roosts internally; E: roosts externally).
